# Comprehensive identification of essential *Staphylococcus aureus *genes using Transposon-Mediated Differential Hybridisation (TMDH)

**DOI:** 10.1186/1471-2164-10-291

**Published:** 2009-07-01

**Authors:** Roy R Chaudhuri, Andrew G Allen, Paul J Owen, Gil Shalom, Karl Stone, Marcus Harrison, Timothy A Burgis, Michael Lockyer, Jorge Garcia-Lara, Simon J Foster, Stephen J Pleasance, Sarah E Peters, Duncan J Maskell, Ian G Charles

**Affiliations:** 1 Department of Veterinary Medicine, University of Cambridge, Madingley Road, Cambridge, CB3 0ES, UK; 2 Arrow Therapeutics, Britannia House, 7 Trinity Street, London, SE1 1DB, UK; 3 Department of Molecular Biology and Biotechnology, University of Sheffield, Firth Court, Western Bank, Sheffield, S10 2TN, UK; 4 Current address: Pfizer Australia, Veterinary Medicine Research and Development, 45 Poplar Road, Parkville, 3052 Victoria, Australia; 5 Current address: Cancer Research Technology Development Laboratory, Wolfson Institute for Biomedical Research, The Cruciform Building, Gower Street, London, WC1E 6BT, UK; 6 Current address: Division of Microbial Diseases, UCL Eastman Dental Institute, 256 Gray's Inn Road, London, WC1X 8LD, UK; 7 Current address: Oxford Gene Technology, Begbroke Science Park, Sandy Lane, Yarnton, Oxford, OX5 1PF, UK; 8 Current address: Centre for Bioinformatics, Division of Molecular Biosciences, Faculty of Natural Sciences, Imperial College London, London, SW7 2AZ, UK

## Abstract

**Background:**

In recent years there has been an increasing problem with *Staphylococcus aureus *strains that are resistant to treatment with existing antibiotics. An important starting point for the development of new antimicrobial drugs is the identification of "essential" genes that are important for bacterial survival and growth.

**Results:**

We have developed a robust microarray and PCR-based method, Transposon-Mediated Differential Hybridisation (TMDH), that uses novel bioinformatics to identify transposon inserts in genome-wide libraries. Following a microarray-based screen, genes lacking transposon inserts are re-tested using a PCR and sequencing-based approach. We carried out a TMDH analysis of the *S. aureus *genome using a large random *mariner *transposon library of around a million mutants, and identified a total of 351 *S. aureus *genes important for survival and growth in culture. A comparison with the essential gene list experimentally derived for *Bacillus subtilis *highlighted interesting differences in both pathways and individual genes.

**Conclusion:**

We have determined the first comprehensive list of *S. aureus *essential genes. This should act as a useful starting point for the identification of potential targets for novel antimicrobial compounds. The TMDH methodology we have developed is generic and could be applied to identify essential genes in other bacterial pathogens.

## Background

There is an urgent need for the development of novel antimicrobial agents to counter the increasing problem of multiply resistant strains of *Staphylococcus aureus *[1]. A first step in the development of new classes of antibiotic is the identification of potential targets within the pathogen genome. Priority targets are genes and gene products that are important for bacterial survival and growth [2]. Several methods of identifying such "essential" genes are described in a recent volume [3]. The most rigorous method is the systematic construction of defined knockout mutants across the whole genome. This has been applied to *Bacillus subtilis *[4], with essential genes defined as those for which a mutant could not be obtained, in many cases being verified by conditional-lethal constructs. However this process is time-consuming and expensive.

An alternative is the use of transposon mutagenesis to generate a library of random mutants. In general, no transposons will be present within essential genes, since the presence of an intragenic transposon will disrupt gene function. The transposon insertion sites can be determined through the use of a transposon-specific primer to amplify the DNA flanking the transposon by PCR. If the library is saturated with a large number of transposon insertions, then essential genes can be identified by genetic footprinting [5]. Alternatively, the primer can be used to sequence the transposon junction directly [6]. However these approaches require a separate PCR and sequencing reaction for every mutated gene; consequently a whole genome screen is again time-consuming and expensive. A higher throughput can be obtained using microarrays to identify simultaneously the location of many inserts [7]; we term these "tag-array" approaches. The regions flanking transposons can be amplified using PCR and hybridised to an amplicon microarray [8], however this is labour intensive and in some cases may be non-reproducible [9]. An alternative is to use a custom transposon with one or two outward-facing promoters, from which labelled RNA run-offs are produced [9]. The use of high-density tiling microarrays can improve resolution [10], but this is still inferior to sequencing-based methods. Small genes (less than ~300 bp) are likely to be problematic since they are only covered by a small number of probes on the array. Large transposon libraries may also be problematic since the signal from a particular probe may be influenced by RNA derived from multiple transposons inserted within the same region.

In order to overcome the limitations of current tag-array approaches we have developed a simple method, Transposon-Mediated Differential Hybridisation (TMDH) [11,12], that combines the advantages of both sequencing- and array-based approaches to determine the repertoire of genes required for the survival and growth of the target organism. Application of TMDH to an analysis of the *S. aureus *genome identified 351 essential genes. Many of these may represent potential targets for the development of new therapeutic approaches to fight this important pathogen. A comparison with the essential gene list of *B*. *subtilis *provides insight into the changes in core metabolism that have occurred since the divergence of the two organisms.

## Results

### Development of the TMDH procedure

**Figure 1:**
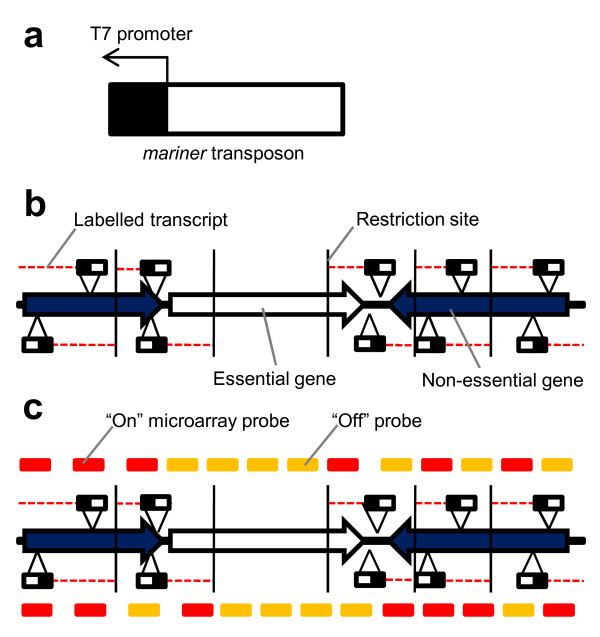
**Diagrammatic representation of the TMDH process**. a) Illustration of the *mariner *transposon b) A saturated genome-wide transposon library is produced. Cells with a transposon disrupting an essential gene will not survive. Genomic DNA from the library is restriction digested, and Cy5-labelled RNA run-offs are produced from the T7 promoters by *in vitro *transcription. c) The labelled RNA is hybridised to a tiling oligonucleotide microarray. Probes that are downstream of a transposon give an "on" signal, other probes give an "off" signal. Note that some probes within an essential gene can give "on" signals, if they can be influenced by transposons outside the gene. Such probes are "non-informative" (see Results and Figure 4). Similarly, probes within a non-essential gene may give an "off" signal if they are upstream of the first transposon.

The TMDH procedure is outlined in Figure 1. Genomic DNA from the TMDH mutant library is digested using an appropriate restriction enzyme and amplified using linker PCR. Transcription is induced from the transposon T7 promoters in the presence of fluorescently-labelled dNTPs, and DNaseI is used to remove DNA, leaving labelled RNA run-offs. The labelled RNA is hybridised to a microarray, and the retrieved data used to screen for the presence of transposons as detailed below. Following the screening process, candidate essential genes are further interrogated for the presence of transposon inserts using PCR and DNA sequencing.

The restriction digest is critical for the TMDH procedure as it provides boundaries to limit the extension of the RNA run-offs, preventing transcripts produced from transposons within non-essential regions from extending into adjacent essential genes. However, for any particular restriction enzyme the distribution of restriction sites may be sub-optimal for some genes, limiting the number of "informative probes" that can be used to assess gene essentiality (see below). To minimise this problem two TMDH experiments were performed, one using the restriction enzyme *Alu*I (AG^CT), the other using *Rsa*I (GT^AC). Even using two enzymes, some genes were still potentially problematic, but sufficiently few that an additional enzyme was not deemed necessary; the affected genes were investigated using PCR (see below).

For each TMDH experiment, chromosomal DNA from the library was digested using the appropriate restriction enzyme, and oligonucleotide linkers were ligated to the restriction fragments. Linker PCR was performed using a linker-specific primer and a transposon-specific primer. *In vitro *transcription was performed from the T7 promoters, with direct incorporation of Cy5-UTP. The reaction was treated with DNaseI and the resultant labelled RNA was purified and used for hybridisation to the microarray.

### Construction of a TMDH transposon library in *S. aureus*

Full details are presented in the Methods section. Briefly, a TMDH transposon, with an outward facing T7 promoter, was initially developed for use in *E. coli *based on the transposon Tn5 (EZ:Tn R6k ori Kan transposon, Epicentre) [12]. The construct was adapted for use in *S. aureus *by the addition of *mariner *mosaic ends (ME) and an erythromycin resistance gene. It was incorporated into a temperature-sensitive (ts) plasmid that contains a chloramphenicol resistance gene and is stable for replication in *S. aureus *at 30°C and below.

**Figure 2:**
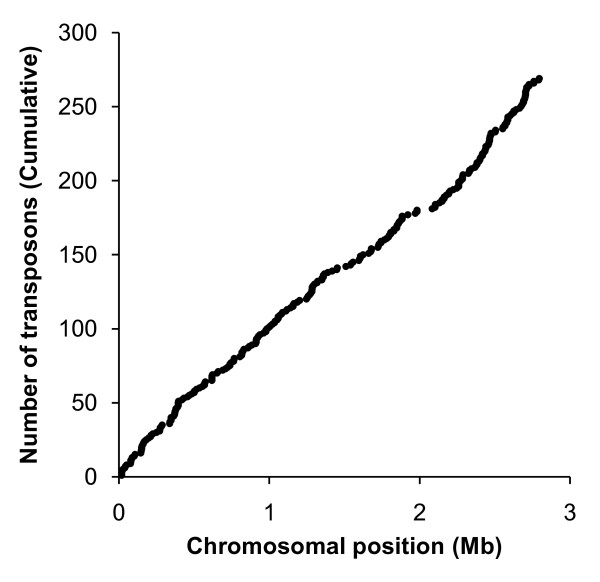
**Cumulative distribution of transposons identified in preliminary experiment**. Transposons were located using either the PCR/sequencing method or an initial microarray-based screen. From these experiments it was concluded that the *mariner *transposon inserted throughout the *S. aureus *chromosome and hence was suitable for use in TMDH.

A large transposon library of around a million mutants was generated in *S. aureus *SH1000, essentially using the procedure described by Bae *et al*. [13]. A serial dilution of the resulting culture was used to determine library size (~10^6 ^mutants) and DNA sequencing and a preliminary TMDH analysis were used to demonstrate that the transposon had integrated throughout the *S. aureus *chromosome (see Figure 2).

### Strain selection

The TMDH method is designed to work for strains for which sequence information can be obtained. This includes non-sequenced strains that are related to strains with a genome sequence. Our experiments were carried out in the *S. aureus *strain SH1000. This was derived from the genome sequenced strain NCTC 8325 (GenBank accession: CP000253) through phage curing followed by reconstruction of the *rsbU *gene [14-16]. The microarray probes that were relevant to TMDH in strain SH1000, and their positions relative to the NCTC 8325 genome sequence, were determined using BLAST (see Methods).

### Microarray procedure

A set of 60-mer oligonucleotide probes was designed based upon the *S. aureus *MW2 genome sequence (GenBank accession: BA000033). The probes were spaced approximately every 100 bases on both strands across the whole genome, with the exception of repetitive regions. In-house inkjet printers capable of generating around 22,000 features per slide were used to produce microarrays [17]. As the total number of probes to span the genome exceeded this capacity, each experiment was performed using three separate slides. Following hybridisation of the labelled RNA and washing, the arrays were scanned using an Agilent G2500A scanner, and the images analyzed using the Agilent Feature Extractor software. Full details of the microarray procedure are available in the Methods section.

### Development of software to determine location of transposon insertions

**Figure 3:**
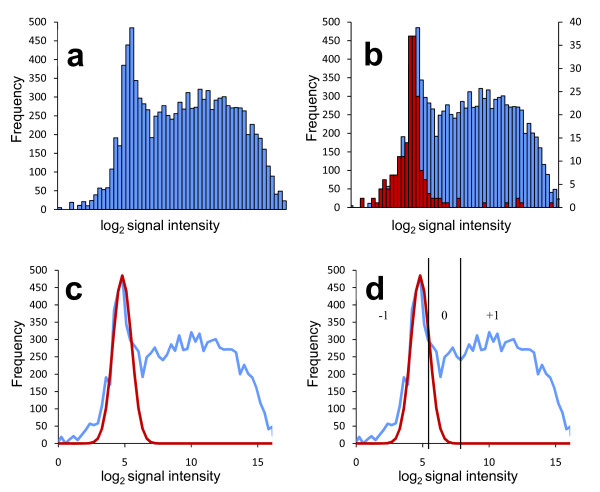
**Illustration of the TMDH microarray scoring system (see text)**. a) Histogram of log_2 _of the raw microarray signals. b) As a), but overlaid with histogram of log_2 _of the microarray signals for the probes which do not hybridise to the *S. aureus *NCTC 8325 genome sequence, and hence indicate the background signal level in the absence of labelled transcript. c) A normal curve is fitted empirically to the data to model the distribution of background signals. d) Cut-offs are defined to classify the probe signals as reflecting the presence (+1) or absence (-1) of transposons. Probes with an intermediate signal are scored as 0, and effectively omitted from the analysis.

For TMDH the primary interest is in a discrete binary property, *i.e*. the presence or absence of transposons. Microarray data are continuously variable, so it is necessary to adopt a strategy for scoring transposons as present or absent. The log_2 _of the spot intensities measured from each microarray show a mixed distribution (see Figure 3a). In the absence of any transposons, low signal intensities with an approximately normal distribution would be expected ("off" signals). In contrast, "on" signals would be expected to have higher intensities but follow an irregular distribution, reflecting the multiple factors that influence the signal if transposons are present. These include the number of transposons present, their distance from probes, and local sequence elements that may affect transcription from the promoter located in the transposon.

To test our hypothesis that the low intensity signals represent the "off" distribution, we exploited the differences between the genomes of the *S. aureus *strains SH1000, in which the library was constructed, and MW2, the genome sequenced strain used to design the microarray probes. Probes designed to hybridise to MW2-specific genomic islands will not hybridise to any target from the SH1000 genome, so act as negative controls. Plotting the data from these probes demonstrates that the lower region of the full distribution corresponds to the signal produced in the absence of any specific hybridisation (Figure 3b). The presence of a small number of higher signals within the negative control dataset suggests some non-specific hybridisation. This issue is addressed in the microarray analysis method detailed below.

For the analysis of TMDH data we developed a method for determining cut-off values to distinguish the "on" and "off" signals. The method is analogous to one commonly used in the analysis of microarray data derived from comparative genome hybridisation [CGH, also referred to as genomotyping; [18]]. The "off" distribution is modelled using a normal distribution fit empirically to the microarray data. A low cut-off point is defined at the point where the number of probes predicted to show that particular signal by the fitted distribution drops below the observed number. A high cut-off is defined at the point where the fitted distribution explains close to 0% of the observed data at that intensity. Probes that gave a signal above the high cut-off were assigned a score of +1, since they were likely to be influenced by the presence of transposons. Probes with a signal below the lower cut-off were given a score of -1, as it is likely that their signal represents the background level without any influence from the RNA produced from the transposon promoters. Probes with an intermediate score were assigned a score of 0, since it was not possible to infer unambiguously the presence or absence of transposons. This method accounts for between-array signal variation, since the curve-fitting procedure is carried out independently for each array. It should also be noted that no account is taken of within-array variation. Standard normalisation approaches cannot be applied due to the high proportion of "on" signals within the dataset.

**Figure 4:**
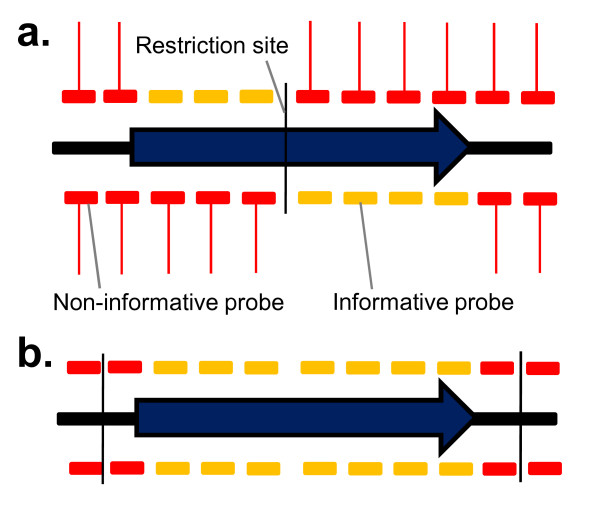
**Procedure for identifying "informative probes" for the automated scoring system**. a) Probes are only informative for a gene if they are downstream of an intragenic restriction site. Other probes may be influenced by transposons located outside the gene, and give an "on" microarray signal (red lines) even if the gene is essential. b) The exception to this rule is if there are no "on" signals anywhere within a restriction fragment. In that case all the probes within the fragment that overlap the gene are informative, since there are no interfering signals.

In TMDH, a probe that hybridises within an essential gene may still give an "on" signal if it is downstream of a transposon that has integrated outside the gene, since RNA run-offs are defined by restriction enzyme sites, and not gene boundaries. Therefore the position of the restriction sites is critical. A method was developed to score only "informative" probes for each restriction fragment (see Figure 4). Probes were only considered informative if they could not be influenced by a transposon outside of the gene. These included probes downstream of an intragenic restriction site (shown in Figure 4 as vertical black lines). If no transposon was evident anywhere within a particular restriction fragment, then all probes from within that fragment were considered informative, since there could be no interference from outside the gene.

To determine a list of candidate essential genes in an automated manner, we examined the score of informative probes overlapping each gene, across all arrays using both restriction enzymes. A simple sum of the scores was found to be the most robust indicator of the presence/absence of transposons. This exploits the presence of multiple probes per gene to minimize the impact of any aberrant signals due to non-specific hybridisation or the lack of normalisation. Genes with a total score of -4 or lower were automatically classified as essential. This cut-off was chosen empirically to minimize the number of false positive genes that were considered unlikely to be essential based on their annotation or prior experimental evidence, whilst retaining most known essential genes. However, the cut-off also results in the omission of genes that had fewer than 4 informative probes, due to their short length or the distribution of restriction sites. The R scripts used to implement the TMDH scoring system are available upon request.

### PCR-based identification of essential genes

**Figure 5:**
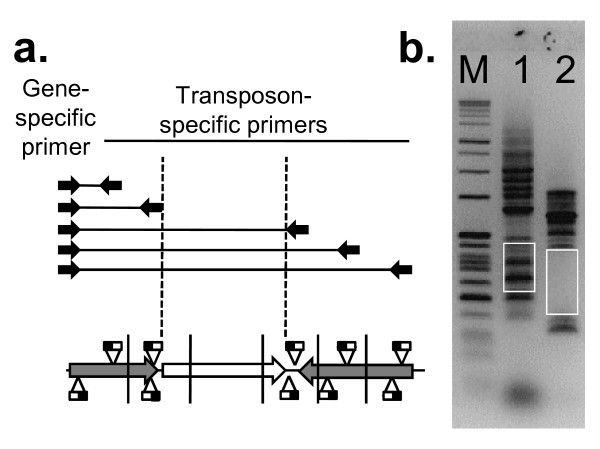
**PCR footprinting strategy used to confirm or reject putative essential genes**. a) Diagrammatic representation of the strategy. PCR is performed using a gene specific primer 50–300 bases upstream of the start codon, and a primer corresponding to the transposon sequence. b) Agarose gel showing sample results. M – size markers, lane 1 – SAOUHSC_00862 (non-essential), lane 2 – SAOUHSC_01672 (essential). Each band represents a PCR product of a different size, corresponding to a transposon insertion in a different position. The white boxes represent the product size range expected for transposon insertions within the gene. For essential genes this region does not contain bands, since cells with insertions in this region are not viable.

Since the automated analysis of microarray data was not expected to be comprehensive, we devised a complementary PCR-based footprinting approach to generate a robust list of essential genes (see Figure 5). A PCR primer was designed between 50 and 300 bases upstream of the target gene. An outward-facing primer was also designed based on the *mariner *transposon sequence. These primers can be used to amplify a range of products, each corresponding to one of the transposon inserts in the library. The size of the products can be determined on an agarose gel, and from each of these the location of the insert can be determined. If a gene is essential, no transposons should be found within the boundaries of the gene, so no PCR products should be seen within the corresponding size range. Most of these genes were investigated further by DNA sequencing, and the gene was considered non-essential if any of the PCR products was confirmed to be derived from an intragenic transposon. An exception to this rule was made if a single PCR product was identified in the region corresponding to the C-terminal portion of the protein where a transposon insertion was considered unlikely to disrupt functionality. If a PCR product could not be obtained, the ability of the gene-specific primer to generate a PCR product was assessed by linker PCR (see Methods).

Manual inspection of the raw microarray data using an online genome browser () allowed us to identify a number of candidate essential genes (mostly small essential genes that are not detectable by the automated analysis). These genes were investigated using the PCR method. To validate the microarray analysis we also performed the PCR confirmation step on all genes identified as essential by the automated method, and also on all genes not identified as essential in *S. aureus*, but which had an essential orthologue in *B. subtilis *[4].

### *S. aureus *Essential Gene List

Following the preliminary microarray screen and automated analysis, 274 candidate essential *S. aureus *genes were identified. These, together with a further 235 candidates chosen as potentially essential by manual inspection of the microarray data, or because of their presence in the *B. subtilis *essential gene list [4], were further investigated by PCR and sequencing. Following this process, 351 *S. aureus *genes were identified that were not disrupted by transposons and constitute our putative *S. aureus *essential gene list. The genes were classified into the same functional categories as used in the *B. subtilis *study [4], and a summary of the findings is presented in Table 1. Additional file 1 contains a full list of the 351 essential genes in *S. aureus*, together with a comparison with the essential gene list from *B. subtilis *[4] and a number of previously published *S. aureus *"essential gene" studies [13,19-21]. The results are also presented in a more compact form in Additional file 2. The full results of the PCR/sequencing analysis are available in Additional file 3. Unless otherwise stated, gene names below are those from the *B. subtilis *GenBank entry, with the *S. aureus *gene name in parentheses if different. Genes that are unnamed and do not have a *B. subtilis *orthologue are referred to by the *S. aureus *NCTC 8325 systematic nomenclature, with the prefix "SAOUHSC_".

**Table 1:** Tabulation of *S. aureus *essential genes by category.

**Category**	**Number of essential genes in *S. aureus***	**Number of *S. aureus *essential genes with an essential orthologue in *B. subtilis***	**Number of *B. subtilis *essential genes with no essential orthologue in *S. aureus***
**DNA metabolism**	**27**	**22**	**3**
DNA replication	16	16	0
DNA packaging and segregation	11	6	3

**RNA metabolism**	**25**	**14**	**2**
Basic transcription machinery	4	4	0
RNA modification	10	7	2
RNA regulation	11	3	0

**Protein synthesis**	**102**	**86**	**7**
Ribosomal proteins	49	47	4
tRNA synthetase	22	20	1
tRNA modification	2	2	0
Translation factors	13	10	1
Protein folding	5	2	0
Protein modification	3	0	0
Protein translocation	8	5	1

**Cell envelope**	**66**	**43**	**22**
Lipids	15	11	0
Phospholipids	4	4	1
Cell wall/amino sugar	3	3	3
Diaminopimelate biosynthesis	0	0	4
Peptidoglycan biosynthesis	17	10	2
Teichoic acid biosynthesis	12	8	0
Cell division	12	7	0
Cell shape	0	0	3
Na/H transporter	0	0	9
Cell envelope – other	3	0	0

**Carbon metabolism**	**20**	**9**	**0**
Glycolysis	11	9	0
Pentose phosphate	4	0	0
Intermediary metabolism	3	0	0
Regulation	2	0	0

**Respiratory pathways**	**11**	**4**	**9**
Isoprenoid/Mevalonate biosynthesis	8	1	2
Menaquinone biosynthesis	1	1	7
Thioredoxin	2	2	0

**Nucleotides**	**12**	**8**	**2**
Purine biosynthesis	3	2	2
Purine metabolism	1	0	0
Purine/Pyrimidine biosynthesis	3	3	0
Pyrimidine biosynthesis	5	3	0

**Cofactors**	**24**	**13**	**2**
Acetyl CoA/CoA	5	1	0
Folate	6	1	2
NAD biosynthesis	4	4	0
SAM	1	1	0
Fe-sulphate cluster	7	6	0
Riboflavin biosynthesis	1	0	0

**Other/unknown**	**64**	**14**	**2**
Amino acid transporter	1	0	0
GTP binding	6	6	0
Other	4	3	1
Unknown	53	5	1

### Influence of experimental conditions on the essential gene list

Several studies have been carried out to determine the minimal set of genes that is essential for growth and replication of a bacterial cell [22-24]. However, any attempt to determine this experimentally will inevitably be influenced by the conditions under which the experiment is performed. A gene may be scored as essential in a particular assay because it is required for survival following exposure to a particular stress inherent in the method, or because it is involved in the uptake or metabolism of the particular nutrients provided in the growth media. An example of this in our method is the requirement for extended incubation of *S. aureus *at 44°C to remove temperature-sensitive replicons. Consequently, genes required for high temperature survival will be scored as putatively essential. To test this, we examined defined mutations in *mrpF*, *dlt*, *tagO*, *fur *and *sarA *(classified as essential from the TMDH score) and all were shown to confer heat sensitivity in *S. aureus *(data not shown). Furthermore, mutations in *clpP *and *dnaK *can cause a growth defect at high temperatures [25,26].

Transposon insertions may not be tolerated within a particular gene even if it is not truly "essential", since they may have a polar effect on the function of a downstream essential gene within the same operon. Examples of potential polarity issues include *rimM *and *recU *(possibly polar on *trmD *and *pbp2*, respectively). Under our experimental conditions, false positives may also be identified if transposon mutagenesis results in a reduced growth rate, since such mutants may be out-competed within the pool. Conversely, false negatives are possible since it may be possible to insert a transposon close to the 3' end of some essential genes without impairing gene function. Insertions close to the 5' end may also be possible if a suitable alternative initiation codon is available. This can also be considered an advantage of the technique, since the transposon screen effectively acts as a truncation assay that gives an indication of the minimal portion of the gene sequence required for function.

### Evaluation of the microarray method as a screen

**Figure 6:**
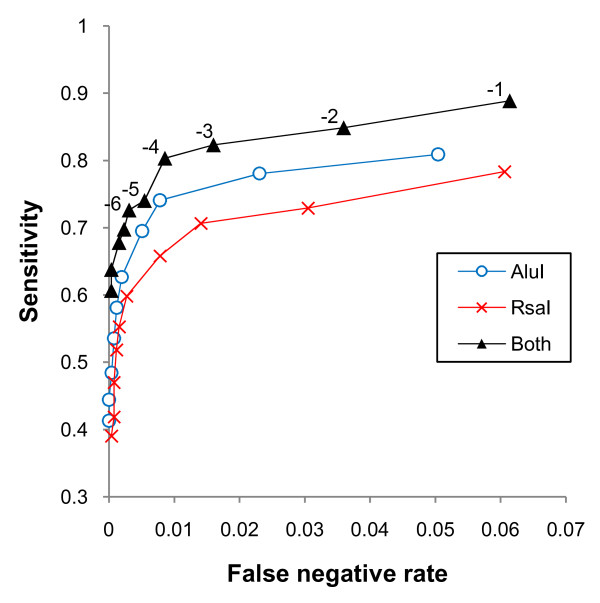
**ROC curves showing sensitivity against false positive rate (1-specificity) for the TMDH microarray screens using individual restriction enzymes, and the combined data from both, for cut-off values from -1 to -10**.

The microarray procedure alone was not expected to comprehensively identify the complete *S. aureus *essential gene list, but was applied as a screen to reduce the workload for the more robust but laborious PCR-based analysis. However, for some applications an exhaustive essential gene list may not be necessary, so it is useful to evaluate the efficacy of the microarray screen. The robust *S. aureus *essential gene list obtained using PCR footprinting allows the sensitivity (percentage of essential genes identified as such in the screen) and specificity (percentage of non-essential genes correctly identified by the screen) of the microarray procedure to be evaluated. Figure 6 shows a ROC curve of sensitivity against false positive rate (1-specificity) for cut-off values from -1 to -10. The curve suggests that a cut-off value of -4 would give an optimal balance between false positives and false negatives in future studies. This gives a specificity of 80.3%, with a sensitivity of 99.1%.

The use of multiple restriction enzymes for TMDH has theoretical advantages, but increases the expense of the method since it involves an additional set of microarrays. Knowledge of the robust essential gene list allows us to evaluate our use of two restriction enzymes, by investigating the data from each microarray screen separately. The analysis of the individual microarray screens is also shown in Figure 6. In these analyses, a cut-off value of -3 is optimal. The *Alu*I experiment performed better than the experiment using *Rsa*I, with specificities of 74.1% and 70.1%, respectively, but both are inferior to the combined dataset. The choice of whether to use a single or multiple enzymes for future studies will depend on the application and available resources.

## Discussion

### DNA metabolism

The core set of 16 genes involved in DNA replication from the *B. subtilis *essential gene list are all scored as essential in *S. aureus *using TMDH. Of the 9 essential *B. subtilis *genes involved in DNA packaging and segregation, 6 were essential in *S. aureus*. The exceptions were *smc*, *ypuG *(*scpA*) and *ypuH *(*scpB*). The products of these three genes form a complex that is involved in chromosome condensation and segregation [27]. The reason for their apparent dispensability in *S. aureus *is unclear.

The essential *B. subtilis *DNA methylation genes *ydiO *and *ydiP *do not have homologues in *S. aureus*. Five additional genes in this category that were not essential in *B. subtilis *were identified as putatively essential in *S. aureus*: *recU*, *ruvA*, *ruvB*, *yrrK *and *yvcI*. These are all associated with recombination and repair, and could be essential to survive the experimental procedure, and thus may be an artefact of the particular methodology that has been used to generate the transposon library.

### RNA metabolism

The four components of the basic transcription machinery (*rpoA*, *rpoB*, *rpoC *and *sigA*) are all essential in both *B. subtilis *and *S. aureus*. There are eight essential genes involved in RNA modification in *B. subtilis*: *cca*, *cspR*, *rnpA*, *rnc*, *trmU*, *trmD*, *ykqC *and *yqjK*. All of these are also essential in *S. aureus*, with the exception of *rnc*, which encodes a ribonuclease, and *cspR*, which encodes a putative RNA methylase. The *trmU *gene is split into two parts in the NCTC 8325 genome sequence, but this is due to a frameshift that occurs in a run of five consecutive adenine residues and most likely represents a sequencing error, or a consequence of slipped strand mis-pairing; the gene is intact in all other available *S. aureus *genome sequences. Three additional essential RNA modification genes were identified in *S. aureus*, *rimM*, *ymfA *and *thdF*. Insertions in *rimM *may not be tolerated due to polar effects on the essential *trmD *gene. *ymfA *is a paralogue of *ykqC *that is non-essential in *B. subtilis*, but is evidently more important in *S. aureus*. The ThdF protein (also known as TrmE) is a tRNA modification GTPase that is conserved across bacteria and eukaryotes, and is essential in some *Escherichia coli *genetic backgrounds [28]. The three essential *B. subtilis *RNA regulation genes, *yycF *(*vicR*), *yycG *(*vicK*) and *nusA *that are conserved in *S. aureus *are all essential, together with eight additional putative regulators: *glnR*, *sarA*, *rnr*, *yvhJ *(*msrR*), *lexA*, *greA*, *yjbD *and *fur*.

### Protein synthesis

There is no direct experimental evidence that the *B. subtilis *complement of 52 ribosomal proteins is essential, but they are considered as such since the ribosome itself is essential [4]. Most of the ribosomal genes appear essential in *S. aureus*. However, the *rpmG *genes, of which there are two copies in both genomes, can each be mutated and are therefore not individually essential in *S. aureus*. The two *S. aureus *copies are more similar to each other than the two *B. subtilis *genes, both showing more similarity to *B. subtilis rpmGA *than to *rpmGB*. *rpmE *is non-essential in *B. subtilis*, due to the presence of two paralogous copies [29], but essential in *S. aureus*, which possesses only a single copy. The *rplI *and *rpsT *genes are essential in *B. subtilis *but not in *S. aureus*. The reason for this is unclear. *rplK *is not essential in *B. subtilis *but was identified as essential by TMDH, possibly due to its involvement in the heat shock response [30]. *rpsJ *is not listed as essential as it is not annotated in the NCTC 8325 genome, but an ORF is present that is predicted to encode a protein identical to RpsJ from other *S. aureus *strains. It is likely that this is an oversight in annotation and *rpsJ *is identified by TMDH as essential in *S. aureus *NCTC 8325.

Of 24 tRNA synthetases listed as essential in *B. subtilis*, 20 are similarly essential in *S. aureus*. Two others, *glyS *and *glyQ*, which are adjacent in the *B. subtilis *genome and encode the α- and β-subunits of a glycyl-tRNA synthetase, are not conserved at the sequence level between *B. subtilis *and *S. aureus*. However, at the equivalent position within a syntenic region, *S. aureus *has an alternative *glyS *gene that is essential. This encodes a class-II glycyl-tRNA synthetase, similar to that encoded by *Bacillus cereus *[31]. The *thrS *and *thrZ *genes of *B. subtilis *can compensate for the absence of each other [4], but only one copy (*thrS*) is present in *S. aureus*, and as may be predicted this is essential. The *asnS *gene is essential in *B. subtilis *but not in *S. aureus*. *yacA*, a gene originally classified as essential but of unknown function in *B. subtilis *[4], is involved in the modification of tRNA-Ile [32] and is also essential in *S. aureus*

The ten genes identified as essential translation accessory factors in *B. subtilis *are all essential in *S. aureus*. The *S. aureus *list includes three additional genes, homologues of *efp*, *yrvI *and *smpB*. The SmpB protein is associated with tmRNA-mediated rescue of stalled ribosomes, and is important for *B. subtilis *growth at high temperatures [33]. *B. subtilis efp *mutants are viable but defective in sporulation [34], however this gene is essential for protein synthesis in *E. coli *[35]. The *yrvI *(*dtd*) gene in *B. subtilis *lacks a start codon, and cell extracts have not shown any D-Tyr-tRNA^Tyr ^deacylase activity [36], suggesting that the gene is non-functional in *B. subtilis*. The gene is intact in other *Bacillus *genomes, so the inactivation may have occurred during the adoption of *B. subtilis *as a laboratory strain. The gene is conserved across most bacteria and eukaryotes, and has been suggested to counteract the toxicity of D-tyrosine [37].

The two genes essential for protein folding in *B. subtilis*, *groES *and *groEL*, are also essential in *S. aureus*. Three other protein folding genes, *dnaJ*, *dnaK *and *grpE*, are scored as essential in *S. aureus*, however *dnaK *mutants are temperature sensitive [26] so may be viable but unable to survive the procedure for generating the transposon library. A homologue of the essential *B. subtilis *post-translational modification gene *map *is also essential in *S. aureus*, however, it shows more similarity to the non-essential *B. subtilis *gene *yflG*. Deformylation is essential in *B. subtilis*, but the genome contains two genes associated with this process (*def *and *ykrB*), and either can be inactivated singly [4]. In *S. aureus *only one deformylation gene, *ykrB *(*pdf1*) is present, and it is essential. Five of the six *B. subtilis *genes essential for protein translocation are also essential in *S. aureus*. The exception is *prsA*, which encodes a protein involved in extracellular folding of secreted proteins. Three additional protein translocation genes, *sipT *(*spsB*), *secDF *and *yqjG*, are essential in *S. aureus*. In *B. subtilis*, *yqjG *has a paralogue, *spoIIIJ*, that is absent from *S. aureus*; in *B. subtilis *the double knockout is lethal [38]. *sipT *encodes one of four closely related type I signal peptidases in *B. subtilis*, whereas only two are present in *S. aureus *(*spsA *and *spsB*). *spsB*, which is most closely related to *B. subtilis sipT*, is essential [39], and has been targeted for the development of novel antimicrobial agents [40]. SpsA, however, lacks residues essential for catalytic activity and is not essential [39].

### Cell Envelope/Cell Wall and Associated Proteins

Sixteen genes involved in the production of the cell membrane are essential in *B. subtilis *[4]. Of these, only *gpsA*, which encodes an NAD-dependent glycerol-3-phosphate dehydrogenase, is not essential in *S. aureus*. Mutants in this gene in other bacteria are auxotrophic for glycerol [41]. Four additional cell envelope genes are essential in *S. aureus*: *fabH*, *fabI*, *ywpB *and *yuxO*. The first three of these are non-essential in *B. subtilis *due to the presence of a second gene with an overlapping function, *fabHB*, *fabL *and *ycsD*, respectively [42,43,4]. The reason for the essential nature of *yuxO*, a possible thioesterase [44], is unclear. Three additional genes that are thought to encode membrane-associated proteins, *pbpX *(*fmtA*), *yfiX *(*fmtC*) and *ypbE *(*ebpS*), are also essential in *S. aureus *but not *B. subtilis*. *pbpX *may be involved in resistance to high temperatures. EbpS in *S. aureus *is an elastin binding protein [45] and although a mutant can be isolated it shows a growth defect [46]. The multi-subunit sodium-hydrogen antiporter encoded by the *mrp *(*mnh*) operon is essential in *B. subtilis *but not in *S. aureus*, most likely due to the presence of a paralogous system in the *S. aureus *genome. A transposon-insertion mutant of *mnhD *in *S. aureus *retains halotolerance and loses transmembrane potential during postexponential growth [47], leading to the suggestion that the *mnh *operon is involved in electron transport. It is possible that the two systems have diverged following a duplication event, but retain the ability to functionally compensate for one another.

The essential *B. subtilis *genes involved in amino sugar biosynthesis prior to peptidoglycan polymerization are all also essential in *S. aureus*, with the exception of *yvyH*, which is present as three copies (*capG*, *capP *and *mnaA*) in the *S. aureus *genome. Diaminopimelate biosynthesis is not important in *S. aureus *as it uses lysine in its peptidoglycan. Of the six *B. subtilis *essential genes involved in the process, only four (*asd*, *dapA*, *dapB *and *ykuQ*) are retained in *S. aureus*. These are required for lysine biosynthesis [48] but are not essential. Of the two racemases that convert L-glutamate and L-alanine into the corresponding D-isomers, *racE *is essential in *S. aureus*, but *alr *is present as two copies neither of which is individually essential. The other genes essential for the synthesis of peptidoglycan precursors in *B. subtilis *(*ddl*, *murAA*, *murB*, *murC*, *murD*, *murE*, *murF*, *murG *and *mraY*) are all similarly essential in *S. aureus*. Three *S. aureus *genes absent from *B. subtilis*, *femA*, *femB *and *fmtB *(*femX*), are essential in the formation of the pentaglycine interpeptide bridge, which is characteristic of *S. aureus *peptidoglycan [49]. Also essential in *S. aureus *but not *B. subtilis *are *ponA *(*pbp2*), *yrvJ *(*lytH*) and SAOUHSC_02107. The essential genes involved in teichoic acid biosynthesis in *B. subtilis *are all essential in *S. aureus*. The genes *dltA*, *B*, *C *and *D *are also scored as essential, but this may be because they are part of the sigma X regulon which is associated with survival at high temperatures in *B. subtilis *[50].

Perhaps unsurprisingly, given the morphological differences between the two species, the orthologues of the essential *B. subtilis *cell shape determining genes, *mreC *and *rodA*, are not essential in *S. aureus*, and there is no orthologue of the *mreB *gene. The essential *B. subtilis *gene *ylaN *is involved in the determination of cell shape, with a similar phenotype to *rodA *[51], and is included in this category. Like *rodA *it is not essential in *S. aureus*. The seven essential cell division-related genes described for *B. subtilis *are all essential in *S. aureus*, together with five others: *ylmF*, *ezrA*, *yyaA*, *gidA *and *ypsB*. YlmF can complement the activity of the FtsZ-binding protein FtsA in *B. subtilis*, to the extent that overexpression of YlmF complemented the otherwise lethal *ftsA *null mutant [52]. These overlapping roles seem to have diverged in *S. aureus*, since both genes are essential. *ezrA *encodes a negative regulator of FtsZ ring formation, which can be knocked out in *B. subtilis *resulting in multiple FtsZ rings [53]. The nucleoid occlusion protein YyaA (also known as Noc) is essential for cell division in the absence of the Min system in *B. subtilis*; the genes encoding the Min system are not present in the *S. aureus *genome. *gidA *mutants affect cell division in *E. coli *cells grown on glucose [54], and GidA may be involved in tRNA modification [55]. It is also essential in *Helicobacter *[56]. YpsB localizes to the cell division site in *B. subtilis*, but its role is unclear [57].

### Carbon Metabolism and Respiration

The core essential genes involved in glycolysis in *B. subtilis *are also essential in *S. aureus*. Three additional components of the glycolysis pathway are essential in *S. aureus*: *pgi*, *gap*, and *pykA*. The essentiality of these three genes means that all components of the pathway from glucose to pyruvate are essential in *S. aureus*, with the exception of the initial conversion of glucose to glucose-6-phosphate. The pentose phosphate pathway also appears to be more important in *S. aureus *than *B. subtilis*, with the component genes *rpe *(*cfxE*), *zwf*, *yqiI *(*gnd*) and SAOUHSC_02612 all scored as essential. A number of genes that play an intermediary or regulatory role in metabolism are also essential. These are: *sucC*, which encodes the beta subunit of succinyl-CoA synthetase, *glnA*, which encodes glutamine synthetase, *yvcK*, which is of unknown function but is required for growth on Krebs cycle intermediates and carbon sources metabolized by the pentose phosphate pathway [58], the HPr (Ser) kinase/phosphatase gene *hprK*, and the phosphotransferase system component *ptsH*. None of these is essential in *B. subtilis*, although *glnA *is essential in *Salmonella enterica *serovar Typhimurium, *Mycobacterium tuberculosis *and *Haemophilus influenzae *[59]. Interestingly, *glnR*, which encodes a repressor of *glnA*, is also scored as essential. The genes are within an operon, so the essentiality of *glnR *could be due to polar effects on *glnA*.

Perhaps the most striking differences between the essential gene complements of *S. aureus *and *B. subtilis *are in the respiratory pathways. The menaquinone biosynthesis pathway, which is essential in *B. subtilis*, seems to be dispensable in *S. aureus*, with the exception of the final step catalyzed by the *menA *gene product. The isoprenoid pathway is essential in *B. subtilis *but in *S. aureus *it is replaced by the mevalonate pathway, where it is itself essential, as it is in other Gram positive cocci [60].

### Nucleotides and Cofactors

There are 10 essential genes involved in nucleotide biosynthesis in the *B. subtilis *list. Of these 8 are essential in *S. aureus*. *guaB *was described as essential in the *B. subtilis *study, but this was a likely consequence of a lack of guanine in the growth medium [4]: it is not required in *S. aureus*, but the downstream gene that encodes GMP synthase, *guaA*, is essential. *hprT*, which was a surprising inclusion in the *B. subtilis *list, does not play an essential role in *S. aureus*. Three other genes, *relA*, which encodes GTP pyrophosphokinase, *pyrH *(*smbA*), which encodes uridylate kinase, and *thyA*, which encodes thymidilate synthase, were all essential in *S. aureus*. *relA*, which mediates the stringent response, has previously been reported as being essential for *in vitro *growth of *S. aureus *[61].

Of the genes involved in cofactor biosynthesis, those required for the production of NAD and SAM are essential in both *S. aureus *and *B. subtilis*. The genes required for the iron-sulphate cluster in *B. subtilis *are also essential in *S. aureus*, with an additional requirement for ferredoxin encoded by *fer*, which is not essential in *B. subtilis*. The pathways to produce CoA and folate are more divergent. In *B. subtilis*, only the last gene in the CoA biosynthesis pathway was essential, suggesting that the immediate precursor (dephospho-CoA) could be scavenged from the medium [4], but that process does not seem to occur in *S. aureus *under our experimental conditions, since three other genes involved in CoA biosynthesis were essential, *ylbI*, *yloI *and SAOUHSC_02371. These allow the synthesis of CoA from the precursor pantothenate, which is presumably obtained from the growth medium. Similarly, folate is not obtained from the growth medium, so all the genes required for its synthesis (*yciA*, *folB*, *folK*, *folP*, *folC *and *dfrA*) are essential. There are two non-homologous genes in *B. subtilis*, *yciA *and *mtrA*, that encode a type I GTP cyclohydrolase (FolE) that catalyses the initial stages of the pathway from GTP to folate [62]. The *S. aureus *genome contains an orthologue of only one of these, *yciA*, which is essential. The riboflavin biosynthesis gene *ribC *was not identified as essential in *B. subtilis *in Kobayashi *et al*. [4], but had been in an earlier study [63], and is essential in *S. aureus*.

### Genes of unknown function

Sixty-six putatively essential genes without a clear indication of their role have been identified in *S. aureus*. Of these, 14 are also essential in *B. subtilis*. Six members of the family of low molecular mass GTP binding proteins, proposed to have ribosome-associated functions [64], are indispensible in *B. subtilis *and we have found them also to be essential in *S. aureus*. Two of the genes, *yneS *and *yfnI *have recently been functionally characterised. YneS (subsequently renamed to PlsY) is a glycerol-3-phosphate acyl transferase required for fatty acid and phospholipid biosynthesis and is essential in *B. subtilis *[65]. YfnI (renamed to LtaS) is essential for lipoteichoic acid biosynthesis in *S. aureus *but not in *B. subtilis *due to the presence of numerous paralogues [66]. Elucidation of the function of further unknowns will shed light on important facets of cellular physiology and form the basis for potential new antibiotic targets.

## Conclusion

To aid in the selection of targets for drug discovery, we have developed TMDH, a method for the genome-wide identification of genes essential for survival and growth *in vitro*. An advantage of our system over other tag-array methods is the software developed for analysis of the microarray data. Use of this automated scoring system allows unbiased prediction of essential genes from even large mutant libraries (in this case a million mutants were used). This method could be applied in isolation to identify potential targets for drug discovery or, as is the case here, used as a preliminary screen to reduce dramatically the workload required for a PCR footprinting and sequencing follow-on strategy to locate unambiguously transposon inserts, and hence infer the essential genes. Our approach incorporates the advantages of tag-array methods, genome-wide coverage and efficiency, and the high resolution associated with PCR/sequencing data.

Application of TMDH to the genome of *S. aureus *allowed the construction of a list of 351 genes that were not disrupted by transposon insertions. It is expected that most of these are essential, although some could be included on the list for other reasons, such as the presence of "cold spot" regions that are refractory to transposon insertion, the possibility of transposon inserts exhibiting polar effects on downstream genes or an inability of some transposon mutants to survive the experimental process. Nevertheless, the list of genes makes biological sense, corresponds well with the previously published non-comprehensive *S. aureus *essential gene lists [13,19-21] and the rigorously determined *B. subtilis *list [4], and should act as a useful starting point for the identification of potential targets for novel antimicrobial compounds. The comparison with the *B. subtilis *essential gene list sheds new light on the biology of both organisms. The major differences are in the respiratory pathways and genes associated with cell morphology, both of which are likely to reflect evolution in different environmental niches. In a number of cases genes that are essential in one organism are non-essential in the other due to the presence of multiple paralogous copies that can presumably compensate for each other. This comparative approach can be used to identify essential processes that are not dependent on a single gene product, but may still represent potential targets for drug development.

TMDH is a powerful technology that can be used for the determination of the essential gene repertoire of a target organism. Our analysis of the *S. aureus *genome is the first such comprehensive study in this organism and highlights the potential of the approach. The application of TMDH to a range of pathogens may highlight important targets for the development of novel antimicrobial therapies. TMDH also has additional potential applications, notably for the identification of conditionally essential genes. One such application has been the use of TMDH to identify *Salmonella enterica *serovar Typhimurium genes required for infection of BALB/c mice [67].

## Methods

### Construction of transposons for TMDH in Gram positive and Gram negative bacteria

A TMDH-compatible transposon was constructed for use in *E. coli *based on Tn*5*. The EZ:Tn R6k ori Kan^r ^transposon (Epicentre) was used as a PCR template with oligonucleotides: 5'-CAGCTGTCTCTTATACACATCTCCCTATAGTGAGTCGTATTACCCATAATACCCATAATAGCTGTTTGCCAgtcgactctagagg-3' and 5'-CAGCTGTCTCTTATACACATCTCTTCTATAGTGTCACCTAAATAGGGATAACAGGGTAATGaattcgttaatacagatgt-3'. These PCR primers introduce a T7 RNA polymerase site with the homing endonuclease site PI-PspI, and an SP6 RNA polymerase site with the homing endonuclease site for I-SceI, respectively. PCR was carried out using a Roche Expand High Fidelity Kit: 96°C for 3 min, then 5 cycles of 96°C for 30 s, 25°C for 90 s and 72°C for 2 min 30 s, then a further 25 cycles at 96°C for 30 s, 50°C for 90 s and 72°C for 2 min 30 s, with a final extension step of 4 minutes followed by cooling to 4°C. The PCR product was separated by gel electrophoresis, and the band corresponding to the expected size of 2044 bp cut out and purified using a gel extraction kit (Qiagen). In order to evaluate the construct, purified transposon DNA was electroporated into 40 μl TransforMax EC100D *pir*^+ ^electrocompetent *E. coli *(Epicentre) using 0.1 cm cuvettes at 200Ω, 25 μF and 20 kV/cm. Following electroporation, cells were grown in 1 ml SOC medium (Gibco Life Technologies) for 1 h at 37°C. Cells were then plated out on L-Agar (Sigma) containing kanamycin at 30 μg/ml (LB-kan). DNA was purified from a panel of colonies and their DNA sequence determined on a Beckman CEQ DNA sequencer (using the manufacturer's recommended conditions) with kan-2-FP-1 (Epicentre) as a DNA sequencing primer. One of the plasmids (pETBlueHGK5) was selected for further study.

### Construction of a *mariner *transposon for TMDH analysis of *S. aureus*

The *mariner *mutagenesis system uses two temperature-sensitive plasmids to introduce transposons into a target strain [13], one bearing the *mariner *transposon (TS1), the other the *mariner *transposase gene (pFA544). The *mariner *plasmids were the kind gift of Dr Fredrik Aslund, Karolinska Institute, Stockholm, Sweden.

In order to construct a *mariner *transposon designed to function in *S. aureus *the following oligonucleotides:

5'-TAACAGGTTGGCTGATAAGTCCCCGGTCTCCCTATAGTGAG-3' and 5'-TAACAGGTTGGCTGATAAGTCCCCGGTCTCTTCTATAGTGTC-3' were used to introduce *mariner *ME ends to the Tn*5 *template described above. The resulting fragment was gel purified (Qiagen), ligated into the *Eco*RV restriction site of pETBlue-1 (Novagen), transformed into *E. coli *and its DNA sequence determined. Clone 5 pETBlueHGK5 was found to contain the expected DNA sequence and was used in future experiments. An erythromycin (*erm*) resistance determinant was amplified from pIL253 [68], using the primers: 5'-GATATCGAAGCAAACTTAAGAGTGT-3' and 5'-GATATCTACAAAAGCGACTCATAGA-3' using standard conditions and cloned into pCR2.1 (Invitrogen). The *erm *cassette was excised from this vector following digestion with *Eco*RV (NEB) purified and ligated into *Hinc*II (NEB) linearised pETBlueHGK5. Following transformation and DNA preparation (Qiagen) the DNA sequence of the resulting construct was determined on a Beckman CEQ DNA sequencer, (using the manufacturer's recommended conditions) and one of the clones (pHGK5erm3) was found to contain the correct sequence. In order to generate a construct that would function in *S. aureus*, plasmid TS1 was digested with the restriction endonuclease *Bam*HI (NEB) and subsequently made blunt-ended by treatment with Klenow fragment (Gibco). The blunt-end fragment was purified and ligated with a *Bgl*II-*Sma*I fragment from pHGK5erm3 comprising the *mariner *TMDH transposon cassette that was also treated with Klenow (as above). The ligation mixture was then transformed into *E. coli *PIR1 cells (Epicentre). Following transformation, cells were plated on LB-Kan (30 μg/ml) and incubated overnight at 37°C. DNA-mini-preps (Qiagen) were sequenced on a Beckman CEQ DNA sequencer using the manufacturer's recommended conditions, with the sequencing primers: 5'-AAGATACTGCACTATCAACACACTC-3' and 5'-CTACCCTGTGGAACACCTACATCT-3'. One of the plasmids (pMARGK2b) was found to contain the correct DNA sequence and was used for further studies. This plasmid comprises the TMDH transposon cassette, a temperature sensitive origin of replication for growth in *S. aureus *(stable replication at temperatures of 30°C or below), and a chloramphenicol resistance gene for selection in *S. aureus *at 5 μg/ml.

### Construction of a *S. aureus *TMDH library

The plasmid pMARGK2b was introduced into *S. aureus *RN4220 by electroporation (0.5 μg pMARGK2b: 2.3 kV (0.1 cm cuvettes), 25 μF, 100Ω) using a Gene Pulser (Biorad). Following electroporation, cells were plated out onto Brain Heart Infusion (BHI) agar (Oxoid) containing chloramphenicol and erythromycin at 5 μg/ml. Following successful growth in RN4220, pMARGK2b was transferred to *S. aureus *SH1000 with the transducing bacteriophage phi-11 using standard methods [15]. Plasmid pFA544 carrying the *mariner *transposase gene was subsequently introduced into *S. aureus *SH1000 carrying pMARGK2b by phi-11 transduction. Transductants were propagated on BHI agar containing chloramphenicol, erythromycin and tetracycline (all at 5 μg/ml) at 32°C for 48 h. The resulting colonies were harvested and inoculated into 600 ml of BHI broth containing chloramphenicol, erythromycin and tetracycline (all at 5 μg/ml) and incubated overnight at 30°C. Following growth, bacteria (100 ml) were recovered by centrifugation at 4000 *g *for 5 min and resuspended in 5 ml BHI broth containing 50% (v/v) glycerol and stored in 0.5 ml aliquots at -80°C.

In order to generate a *S. aureus *SH1000 TMDH mutant library, 0.5 ml of the glycerol stock was inoculated into 100 ml of BHI broth (at room temperature) containing chloramphenicol, erythromycin and tetracycline (all at 5 μg/ml) and incubated at 37°C until the culture reached an A600 of 0.4. Bacteria from a 30 ml sample of this culture were recovered by centrifugation (4000 *g *for 5 min) and the pellet was resuspended in 600 ml BHI broth containing 5 μg/ml erythromycin (pre-warmed to 44°C) and grown until the culture reached an A600 of 0.4. Bacteria from 30 ml of this culture were recovered by centrifugation at 4000 *g *for 5 min and the pellet resuspended in 600 ml BHI broth containing 5 μg/ml erythromycin at 44°C overnight. 10-fold dilutions were made of the resulting primary library (between 10^-3 ^and 10^-6^) and 100 μl aliquots were plated onto BHI plates containing chloramphenicol, tetracycline or erythromycin (all at 5 μg/ml) and grown at 37°C overnight. The resulting growth pattern demonstrated that the bacteria were sensitive to tetracycline and chloramphenicol whilst maintaining resistance to erythromycin. This suggested that the *mariner *TMDH cassette had successfully transposed into the *S. aureus *SH1000 chromosome and the two temperature-sensitive replicons had been lost. This method generated a TMDH library comprising around 10^6 ^mutants. In order to demonstrate that transposon insertion had occurred throughout the *S. aureus *genome, chromosomal DNA was prepared from a number of colonies and the DNA sequence flanking the transposon insertion site determined using the primer 5'-TAGCCAGTTTCGTCGTTAAATGCCC-3' that binds 310 bases 5' from the end of the transposon. The site of transposon integration was also analyzed for a panel of mutants using the TMDH protocol (see below). DNA sequencing and a preliminary TMDH analysis demonstrated that the transposon had integrated throughout the *S. aureus *chromosome (see Figure 2).

### *In vitro *transcription (IVT) reactions

In order to evaluate the suitability of the novel *mariner *transposon for the TMDH protocol, preliminary *in vitro *transcription (IVT) reactions using both SP6 (SP6 MEGAscript kit; Ambion) and T7 (T7 MEGAshortscript kit, Ambion) were carried out following the manufacturer's recommended conditions. 10 μg of genomic DNA from a TMDH library was digested overnight with a target enzyme and purified on a QIAquick column (Qiagen) using the gel extraction protocol, eluted in 200 μl of water, and analyzed by agarose gel electrophoresis to assess the completeness of digestion. For each reaction, 20 μl digested DNA (~1 μg), was reduced in volume by vacuum drying and incorporated into either SP6 or T7 IVT reactions (Ambion) overnight. RNA from IVT reactions was treated with DNaseI, purified on a MinElute column (Qiagen), eluted with 14 μl of water and quantified by measurement at OD_260_. Transcription was observed from both the SP6 and T7 IVT reactions only when the specific RNA polymerase was included in reactions bearing the cognate RNA polymerase binding site.

IVT reactions for *mariner *SH1000 TMDH experiments were produced from DNA fragments flanking the sites of transposon insertion by a Linker PCR (LPCR) strategy. Template for the LPCR reaction was generated by ligating annealed oligonucleotide linkers to restriction digested genomic DNA. LPCR, using a primer pair corresponding to the transposon and to the linker, permits the specific amplification of DNA flanking the site of transposon insertion including the SP6 or T7 promoter sites. Purified genomic DNA (10 μg) from the TMDH library was digested separately with either *Rsa*I or *Alu*I (NEB), in 400 μl, overnight at 37°C. Digested DNA was then purified using a Gel Extraction Kit (Qiagen) and eluted in 50 μl of water. Restriction digests were analyzed by agarose gel (0.8%, w/v, in TBE) electrophoresis to check for completeness. 100 μM each of complementary oligonucleotide pairs (5'-cgactggacctgga-3' and 5'-gataagcagggatcggaacctccaggtccagtcg-3') were annealed by heating to 95°C for 3 min followed by cooling to room temperature over a 1 hr period. The *Rsa*I or *Alu*I linkers were ligated with restriction digested DNA using standard conditions overnight and were purified using a Gel Extraction Kit (Qiagen). We found that during the linker ligation step linkers may self-ligate, giving rise to background signal in subsequent PCR steps. To reduce this possibility, the DNA samples were re-digested with the appropriate restriction enzyme (i.e. linker-ligated *Rsa*I was re-digested with *Rsa*I while linker-ligated *Alu*I was re-digested with *Alu*I). The DNA was then re-purified using a Gel Extraction Kit column (Qiagen). LPCR amplification of the DNA flanking the transposon insertion site was carried out with HotStarTaq, (Qiagen) using the primer pairs: 5'-ctaccctgtggaacacctacatct-3' and 5'-gataagcagggatcggaacc-3' (for the SP6 end) and 5'-cttcgatgactggcaaacagc-3' and 5'-gataagcagggatcggaacc-3' (for the T7 end). Control PCR reactions were performed using PCR primers alone, as well as reactions omitting template. LPCR conditions were as follows; 95°C for 15 min; 30 cycles of 94°C for 30 s, 55°C for 1 min and 72°C for 2 min; 72°C for 5 min, pause at 4°C. Products from each template (*Alu*I, *Rsa*I, T7 and SP6 ends) were purified using the Qiaquick Gel Extraction Kit (Qiagen). Purified DNA was analyzed on a 0.8% (w/v) agarose gel run in TBE.

### Labelling IVT reactions

IVT reactions were carried out using MEGAscript or MEGAshortscript (Ambion) reagents with direct incorporation of either Cy5-UTP or Cy3-UTP (Perkin Elmer). For the T7 promoter, 3.75 μl 10 mM Cy3-UTP was used with 2 μl buffer (10× Ambion stock); 2 μl each of 75 mM ATP, CTP and GTP, and 1.5 μl of UTP; 1 μl (up to 4.75 μl) of template (10 ug of digested gDNA) and 2 μl of enzyme. For the SP6 promoter, 2.5 μl 10 mM Cy5-UTP was used with 2 μl buffer (10× Ambion stock); 2 μl each of 50 mM ATP, CTP and GTP; 1.5 μl 50 mM UTP; 1 μl (up to 6 μl) template and 2 μl enzyme. The reactions were performed overnight at 37°C in the dark in 20 μl reaction volumes.

Labelled RNA was subsequently treated with DNaseI (according to the Ambion protocol) and purified using RNeasy reagents (Qiagen). The concentration of LPCR-derived labelled RNA was determined using a spectrophotometer (1 AU A_260 _= 40 μg/ml RNA).

### Array hybridisations, washing and scanning

Preliminary whole-genome TMDH experiments showed there was no increase in signal resolution by using both T7 and SP6 IVT reactions (labelled with Cy3 and Cy5 respectively), consequently only T7 (Cy3) labelling was used. However, in preliminary experiments to map the position of a small number of transposons, arrays were hybridised with both Cy3 (T7) and Cy5 (SP6) labelled RNA. 100 μl of labelled RNA was added to the hybridisation solution. This consisted of 46.6 μl 12 × MES, 112.0 μl 5 M NaCl, 62.0 μl formamide, 22.4 μl 0.5 M EDTA (pH 8.0), 56.0 μl 10% Triton-X-100 and 161.0 μl water, giving a total volume of 560 μl.

A stock solution of 1 l of MES buffer (2-(*N*-morpholino) ethanesulphonic acid) was made from 70.4 g 2N-morpholenoethene sulphonic acid (MES free acid) and 193.3 g of MES sodium salt (pH 6.5 – 6.7) and filter sterilized through a 0.2 μM) filter. RNA for each array-hybridisation was made up to 50 μl with water, 50 μl of formamide added and the mix denatured by incubation at 55°C for 5 min. The resulting 100 μl volume was then added to the remaining components (460 μl) of hybridisation solution. Hybridisation solutions were added to each microarray in a Hybridisation Chamber (Agilent) and incubated at 50°C for 18 hr. Post-hybridisation washes of the arrays were carried out at room temperature using two 40 ml SSPE (Sigma)-based solutions of 6 × SSPE, 0.005% N-lauryl sarcosine for 5 min, followed by 0.06 × SSPE, 0.18% PEG-200 for 5 min. Arrays were then blown-dry using dry-nitrogen and scanned on an Agilent G2500A Scanner. The raw microarray data have been deposited in ArrayExpress (accession number E-MEXP-1588).

### PCR on genomic DNA from the transposon library

Individual PCR experiments were carried out with a gene-specific primer corresponding to a region of genomic DNA 50–300 bp 5' to the ATG start site (see Additional file 3) in conjunction with a transposon-specific primer. PCRs were carried out using HotStarTaq polymerase (Qiagen) using the manufacturer's recommended conditions for the initial set-up (95°C for 15 min) followed by 30 cycles of 95°C for 45 sec, 50°C for 45 sec and 72°C for 2 min, with a final extension step of 72°C for 10 min. PCR was carried out in a 50 μl reaction mix with a final primer concentration of 0.2 μM. Following PCR, fragments were cloned using TOPO cloning reagents (Invitrogen), and the resulting colonies picked. Colony PCRs were performed on the colonies and PCR products of the appropriate sizes were sequenced directly. DNA sequencing was carried out on an ABI 3100 Automated Capillary DNA Sequencer using the manufacturer's recommended conditions either using plasmid DNA or on colony-PCR products using BigDyeTerminator Cycle Sequencing reagents (Applied Biosystems). Full results are shown in Additional file 3.

### Mapping microarray probes to *S. aureus *NCTC 8325 genome

To characterize the differences from the MW2 genome used to design the microarray, the probes were used as query sequences in a blastn search of the NCTC 8325 genome sequence (accession CP000253). A probe was considered to match uniquely the NCTC 8325 genome if the top hit showed >= 80% identity over >= 50% of the length of the probe, and the second hit did not fulfil both of those criteria. Uniquely matching probes were used in the TMDH analysis, with their hybridisation position on the NCTC 8325 genome determined from the BLAST result. Differences between the experimental strain SH1000 and the genome sequenced strain NCTC 8325 were determined by hybridising genomic DNA from SH1000 against the MW2 TMDH microarray. The probes were scored using the trinary method from GACK [18], and only probes that were scored as present were included in the TMDH analysis.

### Linker PCR

For 87 genes the standard protocol failed to generate PCR product. Typically, this may be due to either problems with the gene-specific primer (e.g. mis-synthesis or sequence error), or the lack of transposons within a specific genomic region suitable for generating a PCR product (e.g. the transposon-specific primer is too far away from the gene-specific primer to generate PCR product under the conditions used). In order to determine whether the gene-specific primer was able to generate PCR product, linker-PCR experiments were carried out using a linker that effectively introduced a PCR primer binding site proximal to the gene-specific primer. Linkers (5'-gataagcagggatcggaacctccaggtccagtcg-3' and 5'-cgactggacctgga-3') were annealed as described above. Genomic DNA from the TMDH library was digested with a 4-base pair blunt-end restriction endonuclease (either *Alu*I (AG^↓^CT) or *Rsa*I (GT^↓^AC)). The restriction enzyme selection was based on a bioinformatic analysis of the DNA sequence of the target gene and chosen to be around 500–1000 bp away from the gene-specific primer-binding site. Blunt-end ligation of the linker onto either *Alu*I or *Rsa*I fragments resulted in the production of short fragments of DNA that could only be amplified optimally with PCR using a gene-specific primer in combination with the linker-specific primer (5'gataagcagggatcggaacc-3') using HotStarTaq polymerase (Qiagen) conditions described above. As the distance of the gene-specific primer from the selected restriction site was known, the size of the resulting PCR product could be used to determine whether or not the gene-specific primer was functional. Linker-PCR results are included in Additional file 3.

### Comparison with other published essential gene lists

Previous studies have identified many *S. aureus *genes as putatively essential [13,19-21]. As these were identified in a variety of strains, the equivalent proteins encoded by strain NCTC 8325 were identified by a blastp search, with an E-value cut-off of 0.001. BLAST results and *x*BASE genome alignments [69] were inspected manually to determine the most likely orthologue if multiple hits were obtained. The NCTC 8325 homologues of genes from the *B. subtilis *essential gene list [4] were determined using the same method. In the case of the *S. aureus *list published by Ji *et al*. [20], the published format used an obsolete nomenclature, and only 138 of their 168 putative essential genes could be identified. Forsyth *et al*. [19] did not publish their essential list in full, only those genes with homologues in *Mycoplasma genitalium *were named and it is this subset of their data that is included here.

## Authors' contributions

IGC & DJM invented the TMDH approach [11]. IGC, DJM, AGA & PJO developed the transposons [12]. IGC, DJM, SJP, SEP, ML, AGA & PJO designed the experiments. AGA, PJO, GS, ML, SJP, JG-L, SJF & SEP performed the experiments. MH and TAB designed the arrays, MH, TAB and RRC designed the PCR primers and KS synthesized arrays. RRC designed the software for the TMDH analysis and RRC, AGA, PJO, GS, MH, TAB, JG-L, SJF, DJM & IGC analyzed the data. RRC, AGA, JG-L, SJF, DJM & IGC wrote the paper. All authors read and approved the final manuscript. In the opinion of the authors RRC and AGA made an equivalent contribution and should be regarded as joint first authors. Similarly, DJM and IGC should be regarded as joint senior authors.

## Supplementary Material

Additional file 1 **Essential *S. aureus *genes, and comparison with the essential gene complement of *B. subtilis *and previous *S. aureus *essential gene studies**. A comprehensive list of *S. aureus *essential genes identified in this study. The names Bae, Ko, Forsyth and Ji refer to the primary authors of previously published *S. aureus *essential gene studies[13,19-21]. The *B. subtilis *essential gene data is derived from the study of Kobayashi *et al*. [4]. Click here for file

Additional file 2 **Essential *S. aureus *genes, and comparison with the essential gene complement of *B. subtilis***. A compact version of the data presented in Additional file 1, suitable for printing. Click here for file

Additional file 3 **Results of PCR/sequencing analysis and linker PCR**. Full results from the PCR/sequencing analysis used to validate the microarray screen. Column C indicates the position of the PCR primer relative to the start codon of the target gene. Columns F and G indicate the size range of the PCR products that correspond to an intragenic transposon. Column K indicates the location of the insertion as determined from the sequence data (where available). For intragenic inserts the first number is the position of the insert relative to the start of the gene. LPCR indicates that no PCR products could be obtained, but the gene-specific primer was verified by linker PCR. Click here for file
