# Supplementary material for: Comprehensive identification of essential Staphylococcus aureus genes using Transposon-Mediated Differential Hybridisation (TMDH)
Source: BMC Genomics. 2009 Jul 1;10:291. doi: 10.1186/1471-2164-10-291 (PMC2721850; doi:10.1186/1471-2164-10-291)
Supplement: Additional file 2 — Essential S. aureus genes, and comparison with the essential gene complement of B. subtilis. A compact version of the data presented in Additional file 1, suitable for printing. [file 1471-2164-10-291-S2.pdf]

Essential in both
Essential in *S.aureus*, non-essential or absent in *B.subtilis*
Essential in *B.subtilis*, present but non-essential in *S.aureus*

Category	*S. aureus* NCTC 8325 gene name	Product	*S. aureus* MW2 gene name	*S. aureus* Mu50 gene name	*S. aureus* N315 gene name	*B. subtilis* gene name
**DNA metabolism**						
DNA replication	SAOUHSC_00001	chromosomal replication initiator protein DnaA	dnaA	SAV0001	dnaA	dnaA
DNA replication	SAOUHSC_00002	DNA polymerase III, beta subunit	dnaN	SAV0002	dnaN	dnaN
DNA replication	SAOUHSC_00018	replicative DNA helicase	dnaC	SAV0016	dnaC	dnaC
DNA replication	SAOUHSC_00349	bacteriophage L54a, single-stranded DNA binding protein	ssb	SAV0366	ssb	ssb
DNA replication	SAOUHSC_00442	DNA polymerase III, gamma and tau subunits, putative	dnaX	SAV0478	dnaX	dnaX
DNA replication	SAOUHSC_00454	DNA polymerase III, delta prime subunit, putative	holB	SAV0484	holB	holB
DNA replication	SAOUHSC_01179	primosomal protein N	priA	SAV1212	priA	priA
DNA replication	SAOUHSC_01241	DNA polymerase III, alpha subunit, Gram-positive type	polC	SAV1264	polC	polC
DNA replication	SAOUHSC_01470	conserved hypothetical protein	MW1343	SAV1453	SA1286	dnaD
DNA replication	SAOUHSC_01663	DNA primase, putative	dnaG	SAV1562	dnaG	dnaG
DNA replication	SAOUHSC_01690	conserved hypothetical protein	MW1538	SAV1587	SA1415	yqeN
DNA replication	SAOUHSC_01791	conserved hypothetical protein	dnaI	SAV1684	dnaI	dnaI
DNA replication	SAOUHSC_01792	conserved hypothetical protein	dnaB	SAV1685	dnaB	dnaB
DNA replication	SAOUHSC_01811	DNA polymerase III, alpha subunit superfamily	dnaE	SAV1703	dnaE	dnaE
DNA replication	SAOUHSC_02122	DNA ligase, NAD-dependent	lig	SAV1904	lig	ligA
DNA replication	SAOUHSC_02123	ATP-dependent DNA helicase PcrA	pcrA	SAV1905	pcrA	pcrA
DNA packaging and segregation	SAOUHSC_00005	DNA gyrase, B subunit	gyrB	SAV0005	gyrB	gyrB
DNA packaging and segregation	SAOUHSC_00006	DNA gyrase, A subunit	gyrA	SAV0006	gyrA	gyrA
DNA packaging and segregation	SAOUHSC_01204	SMC family, C-terminal domain family	smc	SAV1234	smc	smc
DNA packaging and segregation	SAOUHSC_01222	DNA topoisomerase I	MW1133	SAV1250	SA1093	topA
DNA packaging and segregation	SAOUHSC_01351	DNA topoisomerase IV, B subunit	parE	SAV1354	parE	parE
DNA packaging and segregation	SAOUHSC_01352	DNA topoisomerase IV, A subunit	parC	SAV1355	parC	parC
DNA packaging and segregation	SAOUHSC_01490	DNA-binding protein HU, putative	hu	SAV1473	hu	hbs
DNA packaging and segregation	SAOUHSC_01588	conserved hypothetical protein	MW1448	SAV1494	SA1325	ypuH

Category	*S. aureus* NCTC 8325 gene name	Product	*S. aureus* MW2 gene name	*S. aureus* Mu50 gene name	*S. aureus* N315 gene name	*B. subtilis* gene name
DNA packaging and segregation	SAOUHSC_01589	conserved hypothetical protein	MW1449	SAV1495	SA1326	ypuG
DNA packaging and segregation	SAOUHSC_01750	Holliday junction DNA helicase RuvB	ruvB	SAV1641	ruvB	ruvB
DNA packaging and segregation	SAOUHSC_01751	Holliday junction DNA helicase RuvA	ruvA	SAV1642	ruvA	ruvA
DNA packaging and segregation	SAOUHSC_01466	conserved hypothetical protein	recU	SAV1449	recU	recU
DNA packaging and segregation	SAOUHSC_01720	conserved hypothetical protein	MW1566	SAV1616	SA1444	yrrK
DNA packaging and segregation	SAOUHSC_02791	pyrophosphohydrolase, putative	MW2410	SAV2490	SA2278	yvcI

RNA metabolism

Basic transcription machinery	SAOUHSC_00524	RNA polymerase beta chain, putative	rpoB	SAV0542	rpoB	rpoB
Basic transcription machinery	SAOUHSC_00525	DNA directed RNA polymerase beta-prime chain, putative	rpoC	SAV0543	rpoC	rpoC
Basic transcription machinery	SAOUHSC_01662	RNA polymerase sigma factor, putative	sigA	SAV1561	sigA	sigA
Basic transcription machinery	SAOUHSC_02485	DNA-directed RNA polymerase alpha chain, putative	rpoA	SAV2224	rpoA	rpoA

RNA modification	SAOUHSC_01035	conserved hypothetical protein	MW0972	SAV1089	SA0940	ykqC
RNA modification	SAOUHSC_01203	ribonuclease III, putative	rnc	SAV1233	rnc	rnc
RNA modification	SAOUHSC_01209	conserved hypothetical protein	rimM	SAV1239	rimM	rimM
RNA modification	SAOUHSC_01210	tRNA (guanine-N1)-methyltransferase	trmD	SAV1240	trmD	trmD
RNA modification	SAOUHSC_01252	conserved hypothetical protein	MW1158	SAV1275	SA1118	ymfA
RNA modification	SAOUHSC_01474	conserved hypothetical protein	MW1347	SAV1457	SA1290	cca
RNA modification	SAOUHSC_01598	AtsA/ElaC family protein	MW1458	SAV1504	SA1335	yqjK
RNA modification	SAOUHSC_01725	tRNA methyl transferase, putative	MW1571	SAV1621	SA1449	trmU
RNA modification	SAOUHSC_01726	(5-methylaminomethyl-2-thiouridylate)-methyltransferase	MW1571	SAV1621	SA1449	trmU
RNA modification	SAOUHSC_01988	conserved hypothetical protein	MW1796	SAV1855	SA1672	cspR
RNA modification	SAOUHSC_03053	tRNA modification GTPase TrmE	thdF	SAV2712	thdF	thdF
RNA modification	SAOUHSC_03054	ribonuclease P protein component	rnpA		rnpA	rnpA

RNA regulation	SAOUHSC_00020	two-component response regulator, putative	vicR	SAV0018	vicR	yycF
RNA regulation	SAOUHSC_00021	sensory box histidine kinase VicK, putative	vicK	SAV0019	vicK	yycG

Category	*S. aureus* NCTC 8325 gene name	Product	*S. aureus* MW2 gene name	*S. aureus* Mu50 gene name	*S. aureus* N315 gene name	*B. subtilis* gene name
RNA regulation	SAOUHSC_01243	transcription termination-antitermination factor, putative	nusA	SAV1266	nusA	nusA
RNA regulation	SAOUHSC_01592	transcriptional regulator, Fur, putative	MW1452	SAV1498	SA1329	fur
RNA regulation	SAOUHSC_00620	staphylococcal accessory regulator T, putative	sarA	SAV0616	sarA	
RNA regulation	SAOUHSC_00803	ribonuclease R, putative	rnr	SAV0780	rnr	rnr
RNA regulation	SAOUHSC_00934	conserved hypothetical protein	MW0879	SAV0997	SA0856	yjbD
RNA regulation	SAOUHSC_01285	conserved hypothetical protein	glnR	SAV1309	glnR	glnR
RNA regulation	SAOUHSC_01361	transcriptional regulator, putative	msrR	SAV1362	msrR	yvhJ
RNA regulation	SAOUHSC_01333	LexA repressor	lexA	SAV1339	lexA	lexA
RNA regulation	SAOUHSC_01714	transcription elongation factor GreA	greA	SAV1610	greA	greA

Protein synthesis

Ribosomal proteins	SAOUHSC_00017	ribosomal protein L9	rplI	SAV0015	rplI	rplI
Ribosomal proteins	SAOUHSC_00348	ribosomal protein S6	rpsF	SAV0365	rpsF	rpsF
Ribosomal proteins	SAOUHSC_00350	ribosomal protein S18	rpsR	SAV0367	rpsR	rpsR
Ribosomal proteins	SAOUHSC_00518	ribosomal protein L11	rplK	SAV0537	rplK	rplK
Ribosomal proteins	SAOUHSC_00519	ribosomal protein L1	rplA	SAV0538	rplA	rplA
Ribosomal proteins	SAOUHSC_00520	ribosomal protein L10	rplJ	SAV0539	rplJ	rplJ
Ribosomal proteins	SAOUHSC_00521	ribosomal protein L7/L12	rplL	SAV0540	rplL	rplL
Ribosomal proteins	SAOUHSC_00527	ribosomal protein S12	rpsL	SAV0545	rpsL	rpsL
Ribosomal proteins	SAOUHSC_00528	ribosomal protein S7	rpsG	SAV0546	rpsG	rpsG
Ribosomal proteins	SAOUHSC_01078	ribosomal protein L32	rpmF	SAV1128	rpmF	rpmF
Ribosomal proteins	SAOUHSC_01191	ribosomal protein L28	rpmB	SAV1224	rpmB	rpmB
Ribosomal proteins	SAOUHSC_01208	ribosomal protein S16	rpsP	SAV1238	rpsP	rpsP
Ribosomal proteins	SAOUHSC_01211	ribosomal protein L19	rplS	SAV1241	rplS	rplS
Ribosomal proteins	SAOUHSC_01232	ribosomal protein S2	rpsB	SAV1256	rpsB	rpsB
Ribosomal proteins	SAOUHSC_01250	ribosomal protein S15	rpsO	SAV1273	rpsO	rpsO
Ribosomal proteins	SAOUHSC_01328	ribosomal protein L33	rpmG	SAV1335	rpmG	rpmGA
Ribosomal proteins	SAOUHSC_01651	ribosomal protein L33	rpmG	SAV1551	rpmG	rpmGA

Category	*S. aureus* NCTC 8325 gene name	Product	*S. aureus* MW2 gene name	*S. aureus* Mu50 gene name	*S. aureus* N315 gene name	*B. subtilis* gene name
Ribosomal proteins	SAOUHSC_01678	ribosomal protein S21	rpsU	SAV1575	rpsU	rpsU
Ribosomal proteins	SAOUHSC_01689	ribosomal protein S20	rpsT	SAV1586	rpsT	rpsT
Ribosomal proteins	SAOUHSC_01755	ribosomal protein L27	rpmA	SAV1645	rpmA	rpmA
Ribosomal proteins	SAOUHSC_01757	ribosomal protein L21	rplU	SAV1647	rplU	rplU
Ribosomal proteins	SAOUHSC_01784	ribosomal protein L20	rplT	SAV1678	rplT	rplT
Ribosomal proteins	SAOUHSC_01785	ribosomal protein L35	rpmI	SAV1679	rpmI	rpmI
Ribosomal proteins	SAOUHSC_01829	ribosomal protein S4	rpsD	SAV1719	rpsD	rpsD
Ribosomal proteins	SAOUHSC_02361	ribosomal protein L31	rpmE	SAV2120	rpmE	ytiA
Ribosomal proteins	SAOUHSC_02477	ribosomal protein S9, putative	rpsI	SAV2217	rpsI	rpsI
Ribosomal proteins	SAOUHSC_02478	ribosomal protein L13	rplM	SAV2218	rplM	rplM
Ribosomal proteins	SAOUHSC_02484	ribosomal protein L17	rplQ	SAV2223	rplQ	rplQ
Ribosomal proteins	SAOUHSC_02486	ribosomal protein S11, putative	rpsK	SAV2225	rpsK	rpsK
Ribosomal proteins	SAOUHSC_02487	conserved hypothetical protein	rpsM	SAV2226	rpsM	rpsM
Ribosomal proteins	SAOUHSC_02488	ribosomal protein L36	rpmJ	SAV2227	rpmJ	rpmJ
Ribosomal proteins	SAOUHSC_02492	ribosomal protein L15	rplO	SAV2231	rplO	rplO
Ribosomal proteins	SAOUHSC_02493	ribosomal protein L30	rpmD	SAV2232	rpmD	rpmD
Ribosomal proteins	SAOUHSC_02494	ribosomal protein S5	rpsE	SAV2233	rpsE	rpsE
Ribosomal proteins	SAOUHSC_02495	ribosomal protein L18	rplR	SAV2234	rplR	rplR
Ribosomal proteins	SAOUHSC_02496	ribosomal protein L6, putative	rplF	SAV2235	rplF	rplF
Ribosomal proteins	SAOUHSC_02498	ribosomal protein S8, putative	rpsH	SAV2236	rpsH	rpsH
Ribosomal proteins	SAOUHSC_02499	ribosomal protein S14p/S29e, putative	rpsN	SAV2237	rpsN	rpsN
Ribosomal proteins	SAOUHSC_02500	50S ribosomal protein L5, putative	rplE	SAV2238	rplE	rplE
Ribosomal proteins	SAOUHSC_02501	ribosomal protein L24	rplX	SAV2239	rplX	rplX
Ribosomal proteins	SAOUHSC_02502	ribosomal protein L14	rplN	SAV2240	rplN	rplN
Ribosomal proteins	SAOUHSC_02503	30S ribosomal protein S17, putative	rpsQ	SAV2241	rpsQ	rpsQ
Ribosomal proteins	SAOUHSC_02504	ribosomal protein L29	rpmC	SAV2242	rpmC	rpmC
Ribosomal proteins	SAOUHSC_02505	ribosomal protein L16	rplP	SAV2243	rplP	rplP
Ribosomal proteins	SAOUHSC_02506	ribosomal protein S3	rpsC	SAV2244	rpsC	rpsC

Category	*S. aureus* NCTC 8325 gene name	Product	*S. aureus* MW2 gene name	*S. aureus* Mu50 gene name	*S. aureus* N315 gene name	*B. subtilis* gene name
Ribosomal proteins	SAOUHSC_02507	ribosomal protein L22	rplV	SAV2245	rplV	rplV
Ribosomal proteins	SAOUHSC_02508	ribosomal protein S19	rpsS	SAV2246	rpsS	rpsS
Ribosomal proteins	SAOUHSC_02509	ribosomal protein L2	rplB	SAV2247	rplB	rplB
Ribosomal proteins	SAOUHSC_02510	ribosomal protein L23, putative	rplW	SAV2248	rplW	rplW
Ribosomal proteins	SAOUHSC_02511	conserved hypothetical protein	rplD	SAV2249	rplD	rplD
Ribosomal proteins	SAOUHSC_02512	ribosomal protein L3, putative	rplC	SAV2250	rplC	rplC
Ribosomal proteins	SAOUHSC_03055	ribosomal protein L34	rpmH	SAV2714	rpmH	rpmH
Ribosomal proteins	SAOUHSC_00474	ribosomal 5S rRNA E-loop binding protein Ctc/L25/TL5	rplY	SAV0501	rplY	ctc

tRNA synthetase	SAOUHSC_00009	seryl-tRNA synthetase	serS	SAV0009	serS	serS
tRNA synthetase	SAOUHSC_00461	methionyl-tRNA synthetase, putative	metS	SAV0490	metS	metS
tRNA synthetase	SAOUHSC_00493	lysyl-tRNA synthetase	lysS	SAV0517	lysS	lysS
tRNA synthetase	SAOUHSC_00509	glutamyl-tRNA synthetase	gltX	SAV0528	gltX	gltX
tRNA synthetase	SAOUHSC_00511	cysteinyl-tRNA synthetase	cysS	SAV0530	cysS	cysS
tRNA synthetase	SAOUHSC_00611	arginyl-tRNA synthetase	argS	SAV0607	argS	argS
tRNA synthetase	SAOUHSC_00933	tryptophanyl-tRNA synthetase	trpS	SAV0996	trpS	trpS
tRNA synthetase	SAOUHSC_01092	phenylalanyl-tRNA synthetase, alpha subunit	pheS	SAV1138	pheS	pheS
tRNA synthetase	SAOUHSC_01093	phenylalanyl-tRNA synthetase, beta subunit	pheT	SAV1139	pheT	pheT
tRNA synthetase	SAOUHSC_01159	isoleucyl-tRNA synthetase	ileS	SAV1193	ileS	ileS
tRNA synthetase	SAOUHSC_01240	prolyl-tRNA synthetase	proS	SAV1263	proS	proS
tRNA synthetase	SAOUHSC_01471	asparaginyl-tRNA synthetase,putative	asnS	SAV1454	asnS	asnS
tRNA synthetase	SAOUHSC_01666	glycyl-tRNA synthetase	glyS	SAV1565	glyS	
tRNA synthetase	SAOUHSC_01722	alanyl-tRNA synthetase	alaS	SAV1618	alaS	alaS
tRNA synthetase	SAOUHSC_01737	aspartyl-tRNA synthetase	aspS	SAV1630	aspS	aspS
tRNA synthetase	SAOUHSC_01738	histidyl-tRNA synthetase	hisS	SAV1631	hisS	hisS
tRNA synthetase	SAOUHSC_01767	valyl-tRNA synthetase	valS	SAV1663	valS	valS
tRNA synthetase	SAOUHSC_01788	threonyl-tRNA synthetase	thrS	SAV1683	thrS	thrS
tRNA synthetase	SAOUHSC_01839	tyrosyl-tRNA synthetase	tyrS	SAV1729	tyrS	tyrS

Category	*S. aureus* NCTC 8325 gene name	Product	*S. aureus* MW2 gene name	*S. aureus* Mu50 gene name	*S. aureus* N315 gene name	*B. subtilis* gene name
tRNA synthetase	SAOUHSC_01875	leucyl-tRNA synthetase	leuS	SAV1760	leuS	leuS
tRNA synthetase	SAOUHSC_02116	glutamyl-tRNA(Gln) amidotransferase, B subunit	MW1840	SAV1899	SA1715	gatB
tRNA synthetase	SAOUHSC_02117	glutamyl-tRNA(Gln) amidotransferase, A subunit	MW1841	SAV1900	SA1716	gatA
tRNA synthetase	SAOUHSC_02118	glutamyl-tRNA(Gln) amidotransferase, C subunit	MW1842	SAV1901	SA1717	gatC
tRNA Ile modification	SAOUHSC_00484	conserved hypothetical protein	MW0464	SAV0509	SA0467	yacA
tRNA met modification	SAOUHSC_01183	methionyl-tRNA formyltransferase	MW1099	SAV1216	SA1059	fmt
Translation factors	SAOUHSC_00475	peptidyl-tRNA hydrolase	pth	SAV0502	pth	spoVC
Translation factors	SAOUHSC_00529	translation elongation factor G	fus	SAV0547	fus	fusA
Translation factors	SAOUHSC_00530	translation elongation factor Tu	tufA	SAV0548	tufA	tufA
Translation factors	SAOUHSC_00771	peptide chain release factor 2, putative	prfB	SAV0754	prfB	prfB
Translation factors	SAOUHSC_01234	translation elongation factor Ts	MW1140	SAV1257	SA1100	tsf
Translation factors	SAOUHSC_01236	ribosome recycling factor	frr	SAV1259	frr	frr
Translation factors	SAOUHSC_01246	translation initiation factor IF-2	infB	SAV1269	infB	infB
Translation factors	SAOUHSC_01625	translation elongation factor P	MW1481	SAV1528	SA1359	efp
Translation factors	SAOUHSC_01698	conserved hypothetical protein	MW1546	SAV1595	SA1423	yqeI
Translation factors	SAOUHSC_01741	D-tyrosyl-tRNA(Tyr) deacylase	MW1583	SAV1633	SA1459	yrvI
Translation factors	SAOUHSC_01786	translation initiation factor IF-3	infC	SAV1680	infC	infC
Translation factors	SAOUHSC_02359	peptide chain release factor 1	prfA	SAV2118	prfA	prfA
Translation factors	SAOUHSC_02489	translation initiation factor IF-1	infA	SAV2228	infA	infA
Translation factors	SAOUHSC_00804	SsrA-binding protein	ssrP	SAV0782	ssrP	smpB
Protein folding	SAOUHSC_01682	DnaJ protein	dnaJ	SAV1579	dnaJ	dnaJ
Protein folding	SAOUHSC_01683	DNAk protein, putative	dnaK	SAV1580	dnaK	dnaK
Protein folding	SAOUHSC_01684	co-chaperone GrpE	grpE	SAV1581	grpE	grpE
Protein folding	SAOUHSC_02254	chaperonin, 60 kDa, GrpEL, putative	groEL	SAV2029	groEL	groEL

Category	*S. aureus* NCTC 8325 gene name	Product	*S. aureus* MW2 gene name	*S. aureus* Mu50 gene name	*S. aureus* N315 gene name	*B. subtilis* gene name
Protein folding	SAOUHSC_02255	chaperonin, 10 kDa, GroES, putative	groES	SAV2030	groES	groES
Protein modification	SAOUHSC_00790	ATP-dependent Clp protease, proteolytic subunit ClpP	clpP	SAV0768	clpP	clpP
Protein modification	SAOUHSC_02102	methionine aminopeptidase, type I	map	SAV1888	map	yflG
Protein modification	SAOUHSC_01038	polypeptide deformylase	pdf1	SAV1091	pdf1	ykrB
Protein translocation	SAOUHSC_00516	preprotein translocase SecE subunit	secE	SAV0534	secE	secE
Protein translocation	SAOUHSC_00769	preprotein translocase, SecA subunit	secA	SAV0753	secA	secA
Protein translocation	SAOUHSC_00903	Signal peptidase IB, putative	spsB	SAV0965	spsB	sipT
Protein translocation	SAOUHSC_01205	signal recognition particle-docking protein FtsY	MW1118	SAV1235	SA1078	ftsY
Protein translocation	SAOUHSC_01207	signal recognition particle protein	ffh	SAV1237	ffh	ffh
Protein translocation	SAOUHSC_01746	protein-export membrane protein SecDF	secF	SAV1637	secF	secDF
Protein translocation	SAOUHSC_01972	protein export protein PrsA, putative	prsA	SAV1841	prsA	prsA
Protein translocation	SAOUHSC_02327	conserved hypothetical protein	MW2013	SAV2090	SA1893	yqjG
Protein translocation	SAOUHSC_02491	preprotein translocase, SecY subunit, putative	secY	SAV2230	secY	secY

Cell envelope

Lipids	SAOUHSC_00920	conserved hypothetical protein	FabH	SAV0983	FabH	fabHA
Lipids	SAOUHSC_00921	3-oxoacyl- synthase, putative	fab	SAV0984	fab	fabF
Lipids	SAOUHSC_00947	enoyl-(acyl-carrier-protein) reductase	fabI	SAV1011	fabI	fabI
Lipids	SAOUHSC_01197	fatty acid/phospholipid synthesis protein PlsX	plsX	SAV1229	plsX	plsX
Lipids	SAOUHSC_01198	malonyl CoA-acyl carrier protein transacylase	fabD	SAV1230	fabD	fabD
Lipids	SAOUHSC_01199	3-oxoacyl-(acyl-carrier-protein) reductase, putative	fabG	SAV1231	fabG	fabG
Lipids	SAOUHSC_01201	acyl carrier protein	hmrB	SAV1232	hmrB	acpA
Lipids	SAOUHSC_01473	BirA bifunctional protein, putative	MW1346	SAV1456	SA1289	birA
Lipids	SAOUHSC_01623	acetyl-CoA carboxylase, biotin carboxylase	accC	SAV1526	accC	accC
Lipids	SAOUHSC_01624	acetyl-CoA carboxylase, biotin carboxyl carrier protein	accB	SAV1527	accB	accB
Lipids	SAOUHSC_01808	acetyl-CoA carboxylase, carboxyl transferase, alpha subunit	accA	SAV1700	accA	accA

Category	*S. aureus* NCTC 8325 gene name	Product	*S. aureus* MW2 gene name	*S. aureus* Mu50 gene name	*S. aureus* N315 gene name	*B. subtilis* gene name
Lipids	SAOUHSC_01809	acetyl-CoA carboxylase, carboxyl transferase, beta subunit	MW1644	SAV1701	SA1523	accD
Lipids	SAOUHSC_02306	holo-(acyl-carrier-protein) synthase	dpj	SAV2071	dpj	acpS
Lipids	SAOUHSC_02336	beta-hydroxyacyl-ACP dehydratase, putative	MW2023	SAV2098	SA1901	ywpB
Lipids	SAOUHSC_00881	conserved hypothetical protein	MW0826	SAV0944	SA0805	yuxO
Phospholipids	SAOUHSC_01238	conserved hypothetical protein	cdsA	SAV1261	cdsA	cdsA
Phospholipids	SAOUHSC_01260	CDP-diacylglycerol--glycerol-3-phosphate 3-phosphatidyltransferase	pgsA	SAV1283	pgsA	pgsA
Phospholipids	SAOUHSC_01491	glycerol-3-phosphate dehydrogenase, NAD-dependent, putative	gpsA	SAV1474	gpsA	gpsA
Phospholipids	SAOUHSC_01837	1-acyl-sn-glycerol-3-phosphate acyltransferases domain protein	MW1669	SAV1727	SA1548	yhdO
Phospholipids	SAOUHSC_02114	conserved hypothetical protein	MW1839	SAV1898	SA1714	yerQ
Cell wall/amino sugar	SAOUHSC_00120	UDP-N-acetylglucosamine 2-epimerase	cap8G	SAV0155	capG	yvyH
Cell wall/amino sugar	SAOUHSC_00129	UDP-N-acetylglucosamine 2-epimerase	cap8P	SAV0164	capP	yvyH
Cell wall/amino sugar	SAOUHSC_00471	UDP-N-acetylglucosamine pyrophosphorylase	gcaD	SAV0499	gcaD	gcaD
Cell wall/amino sugar	SAOUHSC_02352	UDP-N-acetylglucosamine 2-epimerase	mnaA	SAV2111	mnaA	yvyH
Cell wall/amino sugar	SAOUHSC_02399	glucosamine--fructose-6-phosphate aminotransferase, isomerizing	glmS	SAV2154	glmS	glmS
Cell wall/amino sugar	SAOUHSC_02405	phosphoglucosamine mutase	glmM	SAV2161	glmM(fem D)	ybbT
Diaminopimelate biosynthesis	SAOUHSC_01395	aspartate-semialdehyde dehydrogenase	asd	SAV1394	asd	asd
Diaminopimelate biosynthesis	SAOUHSC_01396	dihydrodipicolinate synthase	dapA	SAV1395	dapA	dapA
Diaminopimelate biosynthesis	SAOUHSC_01397	dihydrodipicolinate reductase	dapB	SAV1396	dapB	dapB
Diaminopimelate biosynthesis	SAOUHSC_01398	2,3,4,5-tetrahydropyridine-2-carboxylate N-succinyltransferase, putative	dapD	SAV1397	dapD	ykuQ
Peptidoglycan biosynthesis	SAOUHSC_00752	UDP-N-acetylenolpyruvoylglucosamine reductase, putative	MW0700	SAV0738	SA0693	murB
Peptidoglycan biosynthesis	SAOUHSC_00954	UDP-N-acetylmuramoylalanyl-D-glutamate--2, 6-diaminopimelate ligase	murE	SAV1018	murE	murE
Peptidoglycan biosynthesis	SAOUHSC_01106	glutamate racemase	murI	SAV1151	murI	racE
Peptidoglycan biosynthesis	SAOUHSC_01146	phospho-N-acetylmuramoyl-pentapeptide- transferase	mraY	SAV1182	mraY	mraY
Peptidoglycan biosynthesis	SAOUHSC_01147	UDP-N-acetylmuramoylalanine--D-glutamate ligase	murD	SAV1183	murD	murD

Category	*S. aureus* NCTC 8325 gene name	Product	*S. aureus* MW2 gene name	*S. aureus* Mu50 gene name	*S. aureus* N315 gene name	*B. subtilis* gene name
Peptidoglycan biosynthesis	SAOUHSC_01237	undecaprenyl diphosphate synthase	uppS	SAV1260	uppS	uppS
Peptidoglycan biosynthesis	SAOUHSC_01400	alanine racemase, putative	MW1287	SAV1399	SA1231	alr
Peptidoglycan biosynthesis	SAOUHSC_01424	UDP-N-acetylglucosamine--N-acetylmuramyl- (pentapeptide) pyrophosphoryl-undecaprenol N-acetylglucosamine transferase	murG	SAV1418	murG	murG
Peptidoglycan biosynthesis	SAOUHSC_01467	penicillin-binding protein 2	pbp2	SAV1450	pbp2	ponA
Peptidoglycan biosynthesis	SAOUHSC_01739	conserved hypothetical protein	MW1582	SAV1632	lytH	yrvJ
Peptidoglycan biosynthesis	SAOUHSC_01856	UDP-N-acetylmuramate--alanine ligase	murC	SAV1740	murC	murC
Peptidoglycan biosynthesis	SAOUHSC_02107	UDP-N-acetylmuramyl tripeptide synthetase, putative	MW1833	SAV1892	SA1708	
Peptidoglycan biosynthesis	SAOUHSC_02305	alanine racemase	alr	SAV2070	alr	alr
Peptidoglycan biosynthesis	SAOUHSC_02317	UDP-N-acetylmuramoylalanyl-D-glutamyl-2, 6-diaminopimelate--D-alanyl-D-alanyl ligase	murF	SAV2082	murF	murF
Peptidoglycan biosynthesis	SAOUHSC_02318	D-alanine--D-alanine ligase	ddlA	SAV2083	ddlA	ddl
Peptidoglycan biosynthesis	SAOUHSC_02337	UDP-N-acetylglucosamine 1-carboxyvinyltransferase	murA	SAV2099	murA	murAA
Peptidoglycan biosynthesis	SAOUHSC_02527	FmhB protein, putative	fmhB	SAV2262	fmhB	
Peptidoglycan biosynthesis	SAOUHSC_01373	methicillin resistance factor, FemA, putative	femA	SAV1374	femA	
Peptidoglycan biosynthesis	SAOUHSC_01374	methicillin resistance factor, putative	femB	SAV1375	femB	

Teichoic acid biosynthesis	SAOUHSC_00223	teichoic acid biosynthesis protein F, putative	MW0230	SAV0254	SA0244	tagF
Teichoic acid biosynthesis	SAOUHSC_00227	conserved hypothetical protein	MW0233	SAV0257	SA0247	tagB
Teichoic acid biosynthesis	SAOUHSC_00640	conserved hypothetical protein	tagA	SAV0636	tagA	tagA
Teichoic acid biosynthesis	SAOUHSC_00641	teichoic acid translocation ATP-binding protein, putative	tagH	SAV0637	tagH	tagH
Teichoic acid biosynthesis	SAOUHSC_00642	teichoic acid biosynthesis protein, putative	tagG	SAV0638	tagG	tagG
Teichoic acid biosynthesis	SAOUHSC_00643	tagB protein, putative	tagB	SAV0639	tagB	tagB
Teichoic acid biosynthesis	SAOUHSC_00645	glycerol-3-phosphate cytidylyltransferase	tagD	SAV0641	tagD	tagD
Teichoic acid biosynthesis	SAOUHSC_00762	conserved hypothetical protein	llm	SAV0747	llm	tagO
Teichoic acid biosynthesis	SAOUHSC_00869	D-alanine-activating enzyme	dltA	SAV0932	dltA	dltA
Teichoic acid biosynthesis	SAOUHSC_00870	dltB protein, putative	dltB	SAV0933	dltB	dltB
Teichoic acid biosynthesis	SAOUHSC_00871	D-alanyl carrier protein	dltC	SAV0934	dltC	dltC
Teichoic acid biosynthesis	SAOUHSC_00872	extramembranal protein	dltD	SAV0935	dltD	dltD

Category	*S. aureus* NCTC 8325 gene name	Product	*S. aureus* MW2 gene name	*S. aureus* Mu50 gene name	*S. aureus* N315 gene name	*B. subtilis* gene name
Cell division	SAOUHSC_00482	conserved hypothetical protein	MW0462	SAV0507	SA0465	divIC
Cell division	SAOUHSC_01144	conserved hypothetical protein	ftsL	SAV1180	ftsL	ftsL
Cell division	SAOUHSC_01154	conserved hypothetical protein	MW1072	SAV1189	SA1032	ylmF
Cell division	SAOUHSC_01149	cell division protein, putative	ftsA	SAV1185	ftsA	ftsA
Cell division	SAOUHSC_01150	cell division protein FtsZ	ftsZ	SAV1186	ftsZ	ftsZ
Cell division	SAOUHSC_01827	conserved hypothetical protein	MW1660	SAV1717	SA1539	ezrA
Cell division	SAOUHSC_03049	conserved hypothetical protein	MW2627	SAV2709	SA2498	yyaA
Cell division	SAOUHSC_01148	cell division protein, putative	div1b	SAV1184	div1b	divIB
Cell division	SAOUHSC_01462	conserved hypothetical protein	MW1335	SAV1446	SA1279	ypsB
Cell division	SAOUHSC_01063	conserved hypothetical protein	MW0996	SAV1113	SA0962	ftsW
Cell division	SAOUHSC_01145	penicillin-binding protein 1	pbpA	SAV1181	pbpA	pbpB
Cell division	SAOUHSC_03052	glucose inhibited division protein A, putative	gidA	SAV2711	gidA	gidA
Cell shape	SAOUHSC_01759	rod shape-determining protein MreC	MW1599	SAV1649	SA1475	mreC
Cell shape	SAOUHSC_02319	conserved hypothetical protein	MW2007	SAV2084	SA1888	rodA
Cell shape	SAOUHSC_01062	conserved hypothetical protein	MW0995	SAV1112	SA0961	ylaN
Na/H transporter	SAOUHSC_00625	conserved hypothetical protein	MW0585	SAV0621	SA0578	mrpA
Na/H transporter	SAOUHSC_00626	conserved hypothetical protein	MW0586	SAV0623	SA0579	mrpB
Na/H transporter	SAOUHSC_00627	conserved hypothetical protein	MW0587	SAV0624	SA0580	mrpC
Na/H transporter	SAOUHSC_00628	conserved hypothetical protein	MW0588	SAV0625	SA0581	mrpD
Na/H transporter	SAOUHSC_00884	Multiple resistance and pH regulation protein F (MrpF / PhaF) family, putative	mnhF	SAV0947	mnhF	mrpF
Na/H transporter	SAOUHSC_00886	Na+/H+ antiporter, MnhD component, putative	mnhD	SAV0949	mnhD	mrpD
Na/H transporter	SAOUHSC_00887	Na+/H+ antiporter subunit, putative	mnhC	SAV0950	mnhC	mrpC
Na/H transporter	SAOUHSC_00888	monovalent cation:proton antiporter subfamily, putative	mnhB	SAV0951	mnhB	mrpB
Na/H transporter	SAOUHSC_00889	Na+/H+ antiporter subunit, putative	mnhA	SAV0952	mnhA	mrpA
Cell envelope - other	SAOUHSC_00998	fmt protein, putative	fmt	SAV1057	fmtA	pbpX

Category	*S. aureus* NCTC 8325 gene name	Product	*S. aureus* MW2 gene name	*S. aureus* Mu50 gene name	*S. aureus* N315 gene name	*B. subtilis* gene name
Cell envelope - other	SAOUHSC_01359	conserved hypothetical protein	fmtC	SAV1360	fmtC	yfiX
Cell envelope - other	SAOUHSC_01501	elastin binding protein	ebpS	SAV1481	ebpS	ypbE
**Carbon metabolism**						
Glycolysis	SAOUHSC_00472	ribose-phosphate pyrophosphokinase, putative	prs	SAV0500	prs	prs
Glycolysis	SAOUHSC_00796	phosphoglycerate kinase, putative	pgk	SAV0773	pgk	pgk
Glycolysis	SAOUHSC_00797	triosephosphate isomerase	tpi	SAV0774	tpi	tpiA
Glycolysis	SAOUHSC_00798	phosphoglycerate mutase, 2,3-bisphosphoglycerate-independent	pgm	SAV0775	pgm	pgm
Glycolysis	SAOUHSC_00799	enolase	eno	SAV0776	eno	eno
Glycolysis	SAOUHSC_00900	glucose-6-phosphate isomerase	pgi	SAV0962	pgi	pgi
Glycolysis	SAOUHSC_01337	transketolase	tkt	SAV1342	tkt	tkt
Glycolysis	SAOUHSC_01806		pykA	SAV1697	pykA	pyk
Glycolysis	SAOUHSC_01807	6-phosphofructokinase, putative	pfk	SAV1698	pfk	pfkA
Glycolysis	SAOUHSC_02366	conserved hypothetical protein	fbaA	SAV2125	fbaA	fbaA
Glycolysis	SAOUHSC_00795	glyceraldehyde-3-phosphate dehydrogenase, type I	gap	SAV0772	gap	gapA
Pentose phosphate	SAOUHSC_01189	ribulose-phosphate 3-epimerase	cfxE	SAV1222	cfxE	rpe
Pentose phosphate	SAOUHSC_01599	glucose-6-phosphate 1-dehydrogenase	MW1459	SAV1505	SA1336	zwf
Pentose phosphate	SAOUHSC_01605	6-phosphogluconate dehydrogenase, decarboxylating	gnd	SAV1511	gnd	yqjI
Pentose phosphate	SAOUHSC_02612	ribose 5-phosphate isomerase, putative	MW2256	SAV2336	SA2127	
Intermediary metabolism	SAOUHSC_01216	succinyl-CoA synthetase, beta subunit, putative	sucC	SAV1245	sucC	sucC
Intermediary metabolism	SAOUHSC_01287	glutamine synthetase, type I	glnA	SAV1310	glnA	glnA
Intermediary metabolism	SAOUHSC_00788	conserved hypothetical protein	MW0728	SAV0766	SA0721	yvcK
Regulation	SAOUHSC_00781	HPr(Ser) kinase/phosphatase	hprK	SAV0760	hprK	hprK
Regulation	SAOUHSC_01028	phosphocarrier protein hpr, putative	ptsH	SAV1083	ptsH	ptsH

Category	*S. aureus* NCTC 8325 gene name	Product	*S. aureus* MW2 gene name	*S. aureus* Mu50 gene name	*S. aureus* N315 gene name	*B. subtilis* gene name
**Respiratory pathways**						
Isoprenoid/Mevalonate biosynthesis	SAOUHSC_00225	conserved hypothetical protein	MW0231	SAV0255	SA0245	yacM
Isoprenoid/Mevalonate biosynthesis	SAOUHSC_00336	acetyl-CoA acetyltransferase, putative	MW0330	SAV0354	SA0342	mmgA
Isoprenoid/Mevalonate biosynthesis	SAOUHSC_00466	4-diphosphocytidyl-2C-methyl-D-erythritol kinase	MW0450	SAV0495	SA0453	ispE
Isoprenoid/Mevalonate biosynthesis	SAOUHSC_00577	mevalonate kinase, putative	mvaK1	SAV0590	mvaK1	
Isoprenoid/Mevalonate biosynthesis	SAOUHSC_00578	diphosphomevalonate decarboxylase	mvaD	SAV0591	mvaD	
Isoprenoid/Mevalonate biosynthesis	SAOUHSC_00579	phosphomevalonate kinase	mvaK2	SAV0592	mvaK2	
Isoprenoid/Mevalonate biosynthesis	SAOUHSC_01618	geranyltranstransferase, putative	ispA	SAV1521	ispA	yqiD
Isoprenoid/Mevalonate biosynthesis	SAOUHSC_02623	conserved hypothetical protein	fni	SAV2346	fni	ypgA
Isoprenoid/Mevalonate biosynthesis	SAOUHSC_02859	hydroxymethylglutaryl-CoA reductase, degradative	mvaA	SAV2545	mvaA	
Isoprenoid/Mevalonate biosynthesis	SAOUHSC_02860	HMG-CoA synthase, putative	mvaS	SAV2546	mvaS	pksG
Menaquinone biosynthesis	SAOUHSC_00980	conserved hypothetical protein	MW0925	SAV1041	SA0894	menA
Menaquinone biosynthesis	SAOUHSC_00983	2-succinyl-6-hydroxy-2, 4-cyclohexadiene-1-carboxylic acid synthase/2-oxoglutarate decarboxylase	menD	SAV1043	menD	menD
Menaquinone biosynthesis	SAOUHSC_00985	enoyl-CoA hydratase/isomerase family protein, putative	menB	SAV1045	menB	menB
Menaquinone biosynthesis	SAOUHSC_01486	heptaprenyl diphosphate syntase component II, putative	gerCC	SAV1470	gerCC	hepT
Menaquinone biosynthesis	SAOUHSC_01487	menaquinone biosynthesis methyltransferase, putative	gerCB	SAV1471	gerCB	menH
Menaquinone biosynthesis	SAOUHSC_01488	conserved hypothetical protein	MW1361	SAV1472	SA1304	hepS
Menaquinone biosynthesis	SAOUHSC_01915	conserved hypothetical protein	menC	SAV1796	menC	menC
Menaquinone biosynthesis	SAOUHSC_01916	o-succinylbenzoic acid--coa ligase, putative	menE	SAV1797	menE	menE
Thioredoxin	SAOUHSC_00785	thioredoxin reductase	trxB	SAV0764	trxB	trxB
Thioredoxin	SAOUHSC_01100	thioredoxin	trxA	SAV1145	trxA	trxA
**Nucleotides**						
Purine biosynthesis	SAOUHSC_00374	inosine-5'-monophosphate dehydrogenase	guaB	SAV0390	guaB	guaB
Purine biosynthesis	SAOUHSC_00375	GMP synthase, putative	guaA	SAV0391	guaA	guaA
Purine biosynthesis	SAOUHSC_00485	hypoxanthine phosphoribosyltransferase	MW0465	SAV0510	SA0468	hprT

Category	*S. aureus* NCTC 8325 gene name	Product	*S. aureus* MW2 gene name	*S. aureus* Mu50 gene name	*S. aureus* N315 gene name	*B. subtilis* gene name
Purine biosynthesis	SAOUHSC_01176	guanylate kinase	gmk	SAV1209	gmk	gmk
Purine biosynthesis	SAOUHSC_02490	adenylate kinase, putative	adk	SAV2229	adk	adk
Purine metabolism	SAOUHSC_01742	GTP pyrophosphokinase	relA	SAV1634	relA	relA
Purine/Pyrimidine biosynthesis	SAOUHSC_00741	ribonucleoside-diphosphate reductase 2, putative	nrdI	SAV0730	nrdI	ymaA
Purine/Pyrimidine biosynthesis	SAOUHSC_00742	ribonucleotide-diphosphate reductase alpha chain, putative	nrdE	SAV0731	nrdE	nrdE
Purine/Pyrimidine biosynthesis	SAOUHSC_00743	ribonucleotide-disphosphate reductase beta chain, putative	nrdF	SAV0732	nrdF	nrdF
Pyrimidine biosynthesis	SAOUHSC_00451	thymidylate kinase	tmk	SAV0482	tmk	tmk
Pyrimidine biosynthesis	SAOUHSC_01235	uridylate kinase, putative	smbA	SAV1258	smbA	pyrH
Pyrimidine biosynthesis	SAOUHSC_01435	thymidylate synthase, putative	thyA	SAV1427	thyA	thyB
Pyrimidine biosynthesis	SAOUHSC_01496	cytidylate kinase	cmk	SAV1478	cmk	cmk
Pyrimidine biosynthesis	SAOUHSC_02368	CTP synthase	ctrA	SAV2127	ctrA	pyrG
**Cofactors**						
Acetyl CoA/CoA	SAOUHSC_00574	phosphate acetyltransferase	pta	SAV0588	pta	pta
Acetyl CoA/CoA	SAOUHSC_01075	pantetheine-phosphate adenylyltransferase	MW1007	SAV1125	SA0973	ylbI
Acetyl CoA/CoA	SAOUHSC_01178	phosphopantothenoylcysteine decarboxylase/phosphopantothenate--cysteine ligase	MW1094	SAV1211	SA1054	yloI
Acetyl CoA/CoA	SAOUHSC_01795	dephospho-CoA kinase	MW1631	SAV1688	SA1511	ytaG
Acetyl CoA/CoA	SAOUHSC_02371	conserved hypothetical protein	MW2054	SAV2130	SA1932	
Folate	SAOUHSC_00490	dihydroneopterin aldolase	folB	SAV0515	folB	folB
Folate	SAOUHSC_00549	conserved hypothetical protein	MW0521	SAV0566	SA0524	yciA
Folate	SAOUHSC_01007	FolD bifunctional protein, putative	folD	SAV1063	folD	folD
Folate	SAOUHSC_01434	dihydrofolate reductase	dfrA	SAV1426	dfrA	dfrA
Folate	SAOUHSC_01766	folylpolyglutamate synthase/dihydrofolate synthase, putative	folC	SAV1662	folC	folC
Folate	SAOUHSC_02354	serine hydroxymethyltransferase, putative	glyA	SAV2113	glyA	glyA

Category	*S. aureus* NCTC 8325 gene name	Product	*S. aureus* MW2 gene name	*S. aureus* Mu50 gene name	*S. aureus* N315 gene name	*B. subtilis* gene name
Folate	SAOUHSC_00491	2-amino-4-hydroxy-6-hydroxymethyldihydropteridine pyrophosphokinase	folK	SAV0516	folK	folK
Folate	SAOUHSC_00489	dihydropteroate synthase	folP	SAV0514	folP	sul
NAD biosynthesis	SAOUHSC_00943	ATP-NAD kinase, putative	MW0888	SAV1007	SA0865	yjbN
NAD biosynthesis	SAOUHSC_01697	nicotinate (nicotinamide) nucleotide adenylyltransferase	MW1545	SAV1594	SA1422	yqeJ
NAD biosynthesis	SAOUHSC_02132	NAD+ synthetase	nadE	SAV1912	nadE	nadE
NAD biosynthesis	SAOUHSC_02133	nicotinate phosphoribosyltransferase, putative	MW1854	SAV1913	SA1729	yueK
SAM	SAOUHSC_01909	S-adenosylmethionine synthetase	metK	SAV1790	metK	metK
Fe-sulpate cluster	SAOUHSC_00847	ABC transporter, ATP-binding protein, putative	MW0795	SAV0842	SA0774	yurY
Fe-sulpate cluster	SAOUHSC_00848	conserved hypothetical protein	MW0796	SAV0843	SA0775	yurX
Fe-sulpate cluster	SAOUHSC_00849	aminotransferase, class V superfamily, putative	MW0797	SAV0844	SA0776	csd
Fe-sulpate cluster	SAOUHSC_00850	conserved hypothetical protein	MW0798	SAV0845	SA0777	yurV
Fe-sulpate cluster	SAOUHSC_00851	conserved hypothetical protein	MW0799	SAV0846	SA0778	yurU
Fe-sulpate cluster	SAOUHSC_01727	conserved hypothetical protein	MW1572	SAV1622	SA1450	yrvO
Fe-sulpate cluster	SAOUHSC_01504	ferredoxin, putative	fer	SAV1484	fer	fer
Riboflavin biosynthesis	SAOUHSC_01249	riboflavin biosynthesis protein RibF	ribC	SAV1272	ribC	ribC
**Other/Unknown**						
Amino acid transporter	SAOUHSC_01787	conserved hypothetical protein	lysP	SAV1681	lysP	rocE
GTP binding	SAOUHSC_01214	conserved hypothetical protein	MW1126	SAV1243	SA1086	ylqF
GTP binding	SAOUHSC_01668	GTP-binding protein Era	bex	SAV1567	bex	era
GTP binding	SAOUHSC_01753	GTP-binding protein	obg	SAV1644	obg	obg
GTP binding	SAOUHSC_01777	GTP-binding protein	MW1617	SAV1673	SA1497	ysxC
GTP binding	SAOUHSC_01700	conserved hypothetical protein	MW1548	SAV1597	SA1425	yqeH

Category	*S. aureus* **NCTC 8325 gene name**	**Product**	*S. aureus* **MW2 gene name**	*S. aureus* **Mu50 gene name**	*S. aureus* **N315 gene name**	*B. subtilis* **gene name**
GTP binding	SAOUHSC_01492	conserved hypothetical protein	MW1364	SAV1475	SA1307	yphC
Other	SAOUHSC_00510	serine acetyltransferase, putative	cysE	SAV0529	cysE	cysE
Other	SAOUHSC_02140	conserved hypothetical protein	MW1860	SAV1919	SA1735	ppaC
Other	SAOUHSC_01416	2-oxoglutarate dehydrogenase, E2 component, dihydrolipoamide succinyltransferase	odhB	SAV1412	odhB	odhB
Other	SAOUHSC_01040	pyruvate dehydrogenase complex, E1 component, alpha subunit, putative	MW0976	SAV1093	pdhA	pdhA
Other	SAOUHSC_02277	O-sialoglycoprotein endopeptidase, putative	MW1973	SAV2049	SA1854	gcp
Unknown	SAOUHSC_00015	conserved hypothetical protein	MW0014	SAV0014	SA0013	yybT
Unknown	SAOUHSC_00226	conserved hypothetical protein	MW0232	SAV0256	SA0246	gutB
Unknown	SAOUHSC_00728	conserved hypothetical protein	MW0681	SAV0719	SA0674	yfnI
Unknown	SAOUHSC_00760	conserved hypothetical protein	MW0708	SAV0746	SA0701	yhcK
Unknown	SAOUHSC_A01041	hypothetical protein				
Unknown	SAOUHSC_01188	conserved hypothetical protein	MW1104	SAV1221	SA1064	yloQ
Unknown	SAOUHSC_01263	conserved hypothetical protein	MW1169	SAV1286	SA1129	ymdA
Unknown	SAOUHSC_01350	conserved hypothetical protein	MW1240	SAV1353	SA1187	yneS
Unknown	SAOUHSC_01477	conserved hypothetical protein	MW1350	SAV1460	SA1293	yugP
Unknown	SAOUHSC_01661	conserved hypothetical protein	MW1512	SAV1560	SA1389	yqfN
Unknown	SAOUHSC_01701	conserved hypothetical protein	MW1549	SAV1598	SA1426	yqeG
Unknown	SAOUHSC_01782	conserved hypothetical protein	MW1621	SAV1677	SA1501	ymaB
Unknown	SAOUHSC_01871	polysaccharide biosynthesis protein, putative	MW1697	SAV1754	SA1575	ytgP
Unknown	SAOUHSC_01908	conserved hypothetical protein	MW1727	SAV1789	SA1607	
Unknown	SAOUHSC_01979	conserved hypothetical protein	MW1788	SAV1847	SA1665	
Unknown	SAOUHSC_02106	conserved hypothetical protein	MW1832	SAV1891	SA1707	
Unknown	SAOUHSC_02151	conserved hypothetical protein	MW1871	SAV1930	SA1744	
Unknown	SAOUHSC_02152	ABC transporter, ATP-binding protein, putative	MW1872	SAV1931	SA1745	yhcH
Unknown	SAOUHSC_02279	conserved hypothetical protein	MW1975	SAV2051	SA1856	ydiC
Unknown	SAOUHSC_02280	conserved hypothetical protein	MW1976	SAV2052	SA1857	ydiB

Category	*S. aureus* NCTC 8325 gene name	Product	*S. aureus* MW2 gene name	*S. aureus* Mu50 gene name	*S. aureus* N315 gene name	*B. subtilis* gene name
Unknown	SAOUHSC_02357	conserved hypothetical protein	MW2040	SAV2116	SA1918	ywlC
Unknown	SAOUHSC_02407	conserved hypothetical protein	MW2090	SAV2163	SA1967	ybbP
Unknown	SAOUHSC_02571	secretory antigen precursor, putative	MW2217	SAV2299	ssaA	
Unknown	SAOUHSC_00003	conserved hypothetical protein	MW0003	SAV0003	SA0003	yaaA
Unknown	SAOUHSC_00444	conserved hypothetical protein	MW0434	SAV0479	SA0437	yaaK
Unknown	SAOUHSC_00575	conserved hypothetical protein	MW0544	SAV0589	SA0546	ywfL
Unknown	SAOUHSC_00793	conserved hypothetical protein	MW0732	SAV0770	SA0725	
Unknown	SAOUHSC_00868	conserved hypothetical protein	MW0813	SAV0931	SA0792	
Unknown	SAOUHSC_00892	conserved hypothetical protein	MW0837	SAV0955	SA0816	yugI
Unknown	SAOUHSC_00922	conserved hypothetical protein	MW0867	SAV0985	SA0844	
Unknown	SAOUHSC_00957	conserved hypothetical protein	MW0902	SAV1022	SA0878	ykoY
Unknown	SAOUHSC_01036	conserved hypothetical protein	MW0973	SAV1090	SA0941	ykzG
Unknown	SAOUHSC_01050	conserved hypothetical protein	MW0986	SAV1103	SA0954	
Unknown	SAOUHSC_01077	conserved hypothetical protein	MW1009	SAV1127	SA0975	ylbN
Unknown	SAOUHSC_01119	conserved hypothetical protein	MW1042	SAV1160	SAS035	
Unknown	SAOUHSC_01190	conserved hypothetical protein	MW1106	SAV1223	SA1066	yloS
Unknown	SAOUHSC_01244	conserved hypothetical protein	MW1150	SAV1267	SA1110	ylxR
Unknown	SAOUHSC_01245	conserved hypothetical protein	MW1151	SAV1268	SA1111	ylxQ
Unknown	SAOUHSC_01627	conserved hypothetical protein	MW1483	SAV1531	SA1361	
Unknown	SAOUHSC_01672	conserved hypothetical protein	MW1522	SAV1570	SA1399	yqfG
Unknown	SAOUHSC_01721	conserved hypothetical protein	MW1567	SAV1617	SA1445	yrzL
Unknown	SAOUHSC_01756	conserved hypothetical protein	MW1596	SAV1646	SA1472	ysxB
Unknown	SAOUHSC_01770	conserved hypothetical protein	MW1610	SAV1666	SAS051	
Unknown	SAOUHSC_01866	conserved hypothetical protein	MW1692	SAV1749	SA1570	ytmP
Unknown	SAOUHSC_01928	transposase family protein, putative	MW1747	SAV0418	SA0379	
Unknown	SAOUHSC_01930	conserved hypothetical protein	MW1748	SAV2476	SA2264	
Unknown	SAOUHSC_02757	conserved hypothetical protein	MW2381	SAV2457	SA2246	
Unknown	SAOUHSC_02805	conserved hypothetical protein	MW2422	SAV2504	SA2292	

Category	*S. aureus* NCTC 8325 gene name	Product	*S. aureus* MW2 gene name	*S. aureus* Mu50 gene name	*S. aureus* N315 gene name	*B. subtilis* gene name
Unknown	SAOUHSC_00345	conserved hypothetical protein	MW0338	SAV0362	SA0350	
Unknown	SAOUHSC_01362	4-oxalocrotonate tautomerase, putative	MW1250	SAV1363	SAS044	ywhB
Unknown	SAOUHSC_02260	delta-hemolysin precursor	hld	SAV2035	hld	
Unknown	SAOUHSC_02572	conserved hypothetical protein	MW2218	SAV2300	SAS083	
Unknown	SAOUHSC_02575	conserved hypothetical protein	MW2221	SAV2303	SA2096	
Unknown	SAOUHSC_02720	conserved hypothetical protein	MW2353			
